# Bisphenol A, nonylphenols, benzophenones, and benzotriazoles in soils, groundwater, surface water, sediments, and food: a review

**DOI:** 10.1007/s11356-014-3974-5

**Published:** 2014-12-30

**Authors:** Alessando Careghini, Andrea Filippo Mastorgio, Sabrina Saponaro, Elena Sezenna

**Affiliations:** DICA - Sezione Ambientale, Politecnico di Milano, Piazza Leonardo da Vinci 32, 20133 Milan, Italy

**Keywords:** Contaminants of emerging concern, Soil, Groundwater, Surface water, Sediments, Food

## Abstract

Contaminants of emerging concern (CECs) are not commonly monitored in the environment, but they can enter the environment from a variety of sources. The most worrying consequence of their wide use and environmental diffusion is the increase in the possible exposure pathways for humans. Moreover, knowledge of their behavior in the environment, toxicity, and biological effects is limited or not available for most CECs. The aim of this work is to edit the state of the art on few selected CECs having the potential to enter the soil and aquatic systems and cause adverse effects in humans, wildlife, and the environment: bisphenol A (BPA), nonylphenol (NP), benzophenones (BPs), and benzotriazole (BT). Some reviews are already available on BPA and NP, reporting about their behavior in surface water and sediments, but scarce and scattered information is available about their presence in soil and groundwater. Only a few studies are available about BPs and BT in the environment, in particular in soil and groundwater. This work summarizes the information available in the literature about the incidence and behavior of these compounds in the different environmental matrices and food. In particular, the review focuses on the physical-chemical properties, the environmental fate, the major degradation byproducts, and the environmental evidence of the selected CECs.

## Introduction

Contaminants of emerging concern (CECs) are defined as any synthetic or naturally occurring chemical that is not commonly monitored in the environment, though having the potential to enter soil and aquatic ecosystems and cause adverse effects in humans, wildlife, and the environment. CECs include synthesized and commercialized chemicals that have just gained entry into the environment and a range of chemicals that have been produced and released into the environment for long, for which new concerns (occurrence, fate, adverse effects on human health and the environment) have recently raised (Focazio et al. 2008). CECs include industrial, agricultural, and household chemicals, such as flame retardants and plasticizers, pesticides, gasoline additives, fluorinated compounds and nanomaterials, as well as pharmaceuticals and personal care products, such as human and veterinary antibiotics and drugs, fragrances, sunscreen agents, antimicrobial cleaning agents, and excipients (Daughton and Ternes 1999; Thomaidis et al. 2012; EUGRIS 2013; Molnar et al. 2013; NORMAN 2013).

CECs can enter the environment from a variety of sources, such as industrial wastes, drain from urbanized areas and transportation systems, sewage treatment plants, atmospheric deposition, etc. (Kolpin et al. 2002; Anderson et al. 2012). On agricultural land, field application of biosolids (manure or sludge) and polymers for modern intensive agriculture (i.e., mulch films, drip irrigation tubes, string, clips, pots, etc.) and irrigation with reclaimed water can be significant sources of CECs (Kolpin et al. 2002; Christian et al. 2003; Kumar et al. 2005). Moreover, due to their continuous release into the environment, these contaminants can accumulate and cause adverse effects in ecosystems, as their transformation/removal rate can be exceeded by their high loading rate (Anderson et al. 2012; USEPA 2014a).

The most worrying consequence of wide use and environmental diffusion of CECs is the increase in the possible exposure pathways for humans, such as ingestion of food plants cultivated on contaminated land or irrigated with reclaimed water, ingestion of meat/animal products from pasture on contaminated land, and consumption of tap water from polluted groundwater or surface water (Weber et al. 2005; Molnar et al. 2013). However, knowledge about the behavior in soil-water systems, toxicity, and biological effects is limited or not available for most CECs; thus, human exposure and related health effects and potential toxicological significance in terrestrial and aquatic ecosystems are mostly unknown and caution is advised. Accordingly, regulatory concentration limits or sound guidance and standard or trigger values for the environmental media have not been established yet (Molnar et al. 2013).

In soil, the behavior of organic contaminants is governed by a variety of complex dynamic physical, chemical, and biological processes, including sorption/desorption, volatilization, leaching, chemical and biological degradation, plant uptake, and runoff (Arias-Estévez et al. 2008). These processes directly control contaminant mobility and fate through the soil and their transfer from soil to water, air, or food. The rate and relative importance of these processes vary with the chemical nature of the contaminant and the chemical, biological, and hydraulic properties of soil (Kibbey et al. 2007). Some compounds, though at trace levels in the sources, accumulate in soils (Kinney et al. 2006; Ternes et al. 2007; Xu et al. 2009), whereas others easily runoff from soil into surface waters or leach to groundwater affecting water reservoirs (Koschorreck et al. 2002). Transport mechanisms related to colloidal material have been underlined for some CECs (Yamamoto and Liljestrand 2003; Zhou et al. 2007).

Most degradation studies were carried out in the aqueous environment (Richardson and Bowron 1985; Buser et al. 1998; Zwiener and Frimmel 2003; Lin and Reinhard 2005; Yu et al. 2006), sewage sludge (Kimura et al. 2007; Zhao et al. 2008), or sediments (Ying and Kookana 2005). Only few studies investigated CEC degradation in soil (Tolls 2001; Gao and Pedersen 2005; Ying and Kookana 2005; Williams and Adamsen 2006; Chefetz et al. 2008; Xuan et al. 2008), showing that some organic contaminants are biodegradable at a certain extent, whereas others exhibit very slow biodegradation rates or are sequestered within soil particles, being inaccessible for microbial degradation. Moreover, most of the previous studies on soil focused on sorption/desorption at equilibrium conditions and degradation under optimal conditions in batch tests, instead of assessing transport dynamics under field conditions (Wehrhan et al. 2007).

The aim of this work is to edit the state of the art on few selected contaminants of emerging concern (bisphenol A, nonylphenol, benzophenones, and benzotriazole) that have been already measured in many European environmental samples and belong to different classes of widely used emerging substances (plasticizers, surfactants, personal care products, and industrial chemicals) (EUGRIS 2013; Molnar et al. 2013; NORMAN 2013). In particular, the review focuses on their physical-chemical properties in relation with their environmental fate and transport, major degradation byproducts, and environmental evidence.

## Bisphenol A (BPA)

Bisphenol A (2,2-bis(4-hydroxyphenyl)propane) is an organic compound composed of two phenol molecules bonded by a methyl bridge and two methyl groups (Table 1).

**Table 1:**
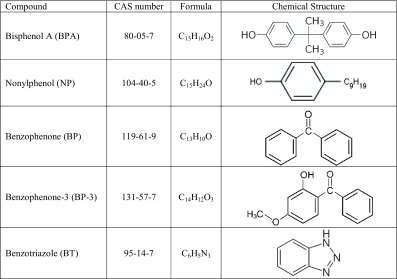
List of chemical compounds studied in the paper

BPA is used as an intermediate (binding, plasticizing, and hardening) in plastics, paints/lacquers, binding materials, and filling materials. Furthermore, it is used as an additive for flame-retardants, brake fluids, and thermal papers. About 95 % of BPA produced in industry is used to make plastics, in particular polycarbonate resins (71 %) and epoxy resins (29 %) (RIKZ 2001; Huang et al. 2012). Due to the increasing demand for polycarbonates and epoxy resins, BPA production has constantly grown in the last years: the global demand was 3.2, 3.9, and 5.0 million tons in 2003, 2006, and 2010, respectively (Flint et al. 2012; Huang et al. 2012).

### Toxicity

BPA is listed as an endocrine disrupter. It has been proven to have estrogenic activity even at concentrations below 1 μg/m^3^ (Rykowska and Wasiak 2006). Estrogenic compounds can have deleterious effects on living organisms because they can disrupt natural hormone balance in both men and women. The effects of exposure to BPA can be particularly harmful to fetus, infants, and young children, because of lack of feedback regulating the activity, synthesis, and elimination of hormones (RIKZ 2001; Rykowska and Wasiak 2006). The acute toxicity of BPA is relatively low. In subacute toxicity studies, a marked reduction in the rate of body weight increase was observed in treated animals (RIKZ 2001). There is limited evidence for carcinogenicity in animals; according to the IARC classification, BPA belongs to group 3 (“not classifiable as to its carcinogenicity to humans”) (IARC 1999).

Ingestion through contaminated food is the major exposure pathway for humans; inhalation and dermal contact are significant exposure pathways for workers involved in the manufacture of BPA. Rykowska and Wasiak (2006) recommended a reference dose (RfD) for oral exposure of 0.01 mg/kg/day. USEPA estimated a reference dose of 50 μg of BPA/kg of body weight/day (USEPA-IRIS 2014).

### Environmental fate and transport

BPA is not produced naturally; it can be released into the environment during production and transport operations, from many products during their use or after their disposal in landfill, through effluent from wastewater treatment plants and from sewage sludge used in agriculture (Huang et al. 2012).

BPA is a moderately water-soluble compound at ambient temperature (Table 2). It has low vapor pressure and does not tend to volatilize significantly from water or dry soil surfaces (Flint et al. 2012). Based on the organic carbon/water partition coefficient (*K*_OC_) value, significant sorption of BPA on soil and sediments is expected. Based on the octanol/water partition coefficient *K*_OW_, BPA has modest capacity for bioaccumulation, which occurs only at high doses (Fent et al. 2003; Flint et al. 2012; Roberts et al. 2014).

**Table 2:** Physical-chemical properties of BPA, 4-NP, BP, BP-3, and BT

Properties	Units	BPA	4-NP	BP	BP-3	BT
Molar weight	g/mol	228.3	220.3	182.2	228.2	119.1
Melting point	°C	158 (RIKZ 2001)	−8 (EC 2002)	49 (Rajendra 2000; Jeon et al. 2006; IARC 2010)	68 (Jeon et al. 2006)	99 (DECOS 2000)
Water solubility (20 °C)	g/m^3^	120–300 (RIKZ 2001; Kalmykova et al. 2013)	6.0 (EC 2002)	Insoluble (Rajendra 2000; IARC 2010)	28.6 (Rodil et al. 2009)	20,000–28,000 (DECOS 2000; Harris et al. 2007)
Henry’s law constant	Pa m^3^/mol	10^−5^–10^−6^ (RIKZ 2001; Fent et al. 2003)	0.84–11.02 (CCME 2002; EC 2002; Soares et al. 2008)	0.19 (IARC 2010)	1.5 × 10^−3^ (Liu et al. 2012a)	1.49 × 10^−2^ (Liu et al. 2011a)
Vapor pressure (25 °C)	Pa	1.1 × 10^−7^–5.3 × 10^−6^ (RIKZ 2001; Kalmykova et al. 2013)	2.07 × 10^−2^–0.3 (EC 2002; Soares et al. 2008)	0.26 (IARC 2010)	7.01 × 10^−4^ (Guidechem 2013)	5.3 (at 20 °C) (TOXNET 2013a)
Octanol-water partition coefficient log(*K*_OW_)	–	2.2–3.4 (RIKZ 2001; Kalmykova et al. 2013)	4.48–5.76 (EC 2002; Soares et al. 2008)	3.18–3.38 (Rajendra 2000; IARC 2010)	3.52–3.82 (Jeon et al. 2006; Sánchez-Brunete et al. 2011; Liu et al. 2012a)	1.23–1.44 (Cornell et al. 2000; Bi et al. 2007; Zhang et al. 2011)
Organic carbon-water partition coefficient log(*K*_OC_)	–	2.5–4.5 (RIKZ 2001; Fent et al. 2003; Flint et al. 2012)	3.4–5.6 (Sekela et al. 1999; Hou et al. 2006)	2.6–2.7 (TOXNET 2013b)	3.43 (Guidechem 2013)	1.27–1.97 (Yu et al. 2009; Liu et al. 2011a)

Hydrolysis is expected to be negligible under environmental conditions due to the absence of hydrolysable groups, but BPA not tied to organic matter undergoes photolysis in water at wavelengths above 290 nm (RIKZ 2001). Pseudo-first-order degradation constants between 0.0053 and 0.008 l/min were observed in aqueous solutions with 10 g/m^3^ of humic substances and BPA between 1 and 20 g/m^3^; no photodegradation occurred in pure water (Zhan et al. 2006).

BPA can be readily biodegraded in soil and sediments under aerobic conditions, with estimated half-life values in soils between 3 and 37.5 days. No degradation was observed in anaerobic soils during 70 day experiments or in anoxic estuarine sediments during 120 day experiments (RIKZ 2001; Fent et al. 2003; Flint et al. 2012; Yu et al. 2013; Chang et al. 2014; Yang et al. 2014). BPA is not expected to be persistent in the environment (USEPA 2010a; Michałowicz 2014).

### Degradation byproducts

Many bacterial strains capable of growing on BPA as a sole source of carbon and energy were isolated from different environmental matrices; they included both gram-negative and gram-positive strains (Zhang et al. 2013a). Different degradation pathways for BPA have been proposed in the literature. In particular, biodegradation of BPA proceeds via complicated metabolic routes that leads to formation of several kinds of byproducts (Spivack et al. 1994; Ike et al. 2002; Zhan et al. 2006; Ye et al. 2011).

Zhan et al. (2006) proposed a photodegradation pathway in aqueous solution with humic substances based on the results of structural analyses for intermediate photoproducts. Mono-hydroxylated BPA, glycerol, 2-hydroxy-propanoic acid, and *p*-hydroquinone were identified as degradation products.

Zhang et al. (2007) studied BPA degradation by a microbial strain isolated from the compost leachate of a municipal solid waste; 4-hydroxybenzaldehyde, 4-hydroxybenzoic acid, and *p*-hydroquinone were the observed metabolic intermediates. Also, Dodgen et al. (2014) detected 4-hydroxybenzaldehyde and 4-hydroxybenzoic acid, together with 4-hydroxyacetophenone, as transformation products of BPA in degradation tests using different artificially contaminated soils.

Spivack et al. (1994) studied the degradation pathway for a gram-negative aerobic bacterium (pure culture). The major route (80 %) was BPA cleavage to *p*-hydroxyacetophenone (*p*-HAP) and *p*-hydroxybenzaldehyde (*p*-HBAL), followed by further degradation via *p*-hydroxybenzoic acid (*p*-HBA). The remaining 20 % of BPA was converted into 2,3-bis-(4-hydroxyphenyl)-1,2-propanediol (tetraol-IV) via bis(4-hydroxyphenyl)-1-propanol. Although tetraol-IV can be slowly degraded to *p*-hydroxyphenacyl alcohol (*p*-HPOH), both these byproducts accumulated in the medium. In Ike et al. (2002), BPA degradation by mixed microbial consortia from activated sludge or river water also led in most cases to the accumulation of the minor route byproducts tetraol-IV and *p*-HPOH, generally identified as the dead-end compounds. Based on the studies reported above, BPA photodegradation and biodegradation do not seem to mineralize the compound.

Ye et al. (2011) investigated BPA metabolism in rat and human liver microsomes. The oxidative metabolism of BPA to BPA catechol was a major pathway when using male rat microsomes, but only a minor pathway (less than 10 % BPA catechol was formed) when using human liver microsomes.

### Environmental evidence

The major studies reported in the literature about BPA presence in environmental matrices and food are reported in Table 3. Industrial activities (mainly chemical plants) and wastewater treatment plants were the major sources of BPA in the surface waters and sediments. High concentrations in soil and groundwater were detected especially for agricultural fields irrigated with treated wastewater and/or amended with biosolids or near landfills (Heemken et al. 2001; Kawahata et al. 2004; Cespedes et al. 2005; Vethaak et al. 2005; Loos et al. 2007; Yoon et al. 2010; Félix-Cañedo et al. 2013; Wu et al. 2013; Gorga et al. 2014; Michałowicz 2014).

**Table 3:** BPA concentrations in various environmental matrices and in food (percentages between brackets represent the detection frequency)

Reference	Location	Units	Value
Soils			
Kinney et al. (2008)	Agricultural fields, USA	μg/kg d.w.	<32–147 Mean 59
Xu et al. (2008)	Golf course irrigated with reclaimed wastewater, California, USA	μg/kg d.w.	0.55–2
Gibson et al. (2010)	Agricultural fields irrigated with wastewater, Tula Valley, Mexico	μg/kg d.w.	1.6–30.2 Mean 8.3
Staples et al. (2010)	Soils amended with biosolids, North America (data collected in the period 1990–2006)	μg/kg d.w.	Median 1.15 95th percentile 21
Staples et al. (2010)	Soils amended with biosolids, Europe (data collected in the period 1990–2006)	μg/kg d.w.	Median 0.24 95th percentile 140
USEPA (2010a)	Range of values in USA	μg/kg d.w.	4–14 Mean 6–7
Sediments			
Heemken et al. (2001)	Elbe River and some of its tributaries, Germany	μg/kg d.w.	66–343 Mean 163
Kawahata et al. (2004)	Estuarine and marine sediments from Okinawa and Ishigaki Islands, Japan	μg/kg d.w.	<0.5–13 Mean 3.2
Vethaak et al. (2005)	Fresh, marine, and estuarine sediments, The Netherlands	μg/kg d.w.	<1.1–43 Median 3.2 (78 %)
Fu et al. (2007)	Estuarine and marine sediments from Jiaozhou Bay and surrounding rivers, China	μg/kg d.w.	0.7–27.3
Pojana et al. (2007)	Sediments from Venice Lagoon, Italy	μg/kg d.w.	<2.0–118 Mean 36
USEPA (2010a)	Fresh sediments, USA	μg/kg d.w.	1.4–140
USEPA (2010a)	Marine sediments, USA	μg/kg d.w.	1.5–5.0
Gorga et al. (2014)	Ebro River basin, Spain	μg/kg d.w.	<0.24–100
Michałowicz (2014)	Elba River sediments, Germany	μg/kg d.w.	10–380
Michałowicz (2014)	16 major rivers’ sediments, Taiwan	μg/kg d.w.	0.37–492
Stewart et al. (2014)	Estuarine sediments from Auckland, New Zealand	μg/kg d.w.	<50–145 Mean 57
Wu et al. (2013)	Huangpu River and its tributaries, China	μg/kg d.w.	0.96–14.44 Mean 7.22
Gorga et al. (2015)	Different rivers, Spain	μg/kg d.w.	<0.24–117
Groundwater			
Lacorte et al. (2002)	Agricultural area in Catalonia, Spain	mg/m^3^	<0.01–0.35
Latorre et al. (2003)	Agricultural areas in northern Spain	mg/m^3^	0.05–0.18
Godejohann et al. (2009)	Ammunition disposal site, Switzerland	mg/m^3^	12–13
Loos et al. (2010)	Survey on European groundwaters	mg/m^3^	<0.001–2.299 Mean 0.079 90th percentile 0.073
USEPA (2010a)	Range of mean values in USA	mg/m^3^	0.004–1.9
Stuart et al. (2011)	Groundwater, England	mg/m^3^	up to 20
Félix-Cañedo et al. (2013)	Groundwater in Mexico City, Mexico	mg/m^3^	<0.0005–0.010 (63 %)
Luo et al. (2014)	Groundwater in Europe	mg/m^3^	Mean 0.079, maximum 2.299
Luo et al. (2014)	Groundwater in USA	mg/m^3^	Mean 2.550
Michałowicz (2014)	Groundwater contaminated with leachate from refuse dump in Osaka, Japan	mg/m^3^	740
Surface water			
Azevedo et al. (2001)	River and coastal waters, Portugal	mg/m^3^	0.07–4.0 Mean 1.0
Heemken et al. (2001)	Elbe River and some of its tributaries, Germany	mg/m^3^	0.017–0.776 Mean 0.105
Basheer et al. (2004)	Surface coastal water, Singapore	mg/m^3^	<0.002–2.47 Mean 0.40
Kawahata et al. (2004)	Estuarine and marine waters from Okinawa, and Ishigaki Islands, Japan	mg/m^3^	<0.005–0.08 Mean 0.02
Cespedes et al. (2005)	Llobregat River basin, Spain	mg/m^3^	<0.09–2.97 Mean 0.44
Vethaak et al. (2005)	Fresh, marine and estuarine water, The Netherlands	mg/m^3^	<0.009–1.0 Median 0.045 (52 %)
Patrolecco et al. (2006)	Tiber River, Italy	mg/m^3^	<0.03–0.14 Mean 0.07
Vousta et al. (2006)	Glatt River, Switzerland	mg/m^3^	0.009–0.076
Fu et al. (2007)	Estuarine and marine water from Jiaozhou Bay, China	mg/m^3^	0.0015–0.262
Loos et al. (2007)	River water, Belgium	mg/m^3^	0.003–0.055 Mean 0.031
Loos et al. (2007)	River water, Italy	mg/m^3^	<0.002–0.175 Mean 0.065
Pojana et al. (2007)	Venice Lagoon, Italy	mg/m^3^	<0.001–0.145 Mean 0.014
Yoon et al. (2010)	Han River, South Korea	mg/m^3^	0.0069–0.059 Mean 0.027
Yoon et al. (2010)	Effluent-dominated creeks discharging into Han River, South Korea	mg/m^3^	0.011–0.120 Mean 0.062
USEPA (2010a)	Range of mean values in USA	mg/m^3^	0.012–0.14
Félix-Cañedo et al. (2013)	Surface water (dams) in Mexico City, Mexico	mg/m^3^	<0.0005–0.007 (52 %)
Esteban et al. (2014)	Manzanares and Jarama rivers, Spain	mg/m^3^	0.006–0.126
Luo et al. (2014)	Canada	mg/m^3^	Mean 0.0021 Maximum 0.087
Luo et al. (2014)	China	mg/m^3^	0.006–0.881
Luo et al. (2014)	Germany	mg/m^3^	0.192–0.215
Luo et al. (2014)	Greece	mg/m^3^	0.055–0.152
Luo et al. (2014)	Korea	mg/m^3^	0.0075–0.334
Luo et al. (2014)	UK	mg/m^3^	0.006–0.068
Michałowicz (2014)	Range of concentrations in rivers, Portugal	mg/m^3^	0.029–0.098
Melo and Brito (2014)	Rivers crossing Sao Luis island, Brazil	mg/m^3^	<0.46
Michałowicz (2014)	Elba River, Germany	mg/m^3^	4–92
Michałowicz (2014)	16 major rivers, Taiwan	mg/m^3^	0.01–45
Wu et al. (2014)	Huangpu River and its tributaries, China	mg/m^3^	0.0071–0.1115 Mean 0.0276
Xu et al. (2014)	Cape D’ Aguilar Marine Reserve, Hong Kong, wet season	mg/m^3^	0.011–0.41 Mean 0.0645
Xu et al. (2014)	Cape D’ Aguilar Marine Reserve, Hong Kong, dry season	mg/m^3^	0.025–0.24 Mean 0.0695
Zhang et al. (2014)	North Tai Lake Basin, Eastern China	mg/m^3^	0.024–1.175 Mean 0.270
Gorga et al. (2015)	Iberian rivers (Ebro, Llobregat, Júcar and Guadalquivir)	mg/m^3^	0.00011–0.649
Food			
Basheer et al. (2004)	Seafood from supermarkets, Singapore	μg/kg f.w.	13.3–213.1 Mean 82.5
Sun et al. (2006)	Canned vegetables, fruits, and meats from local supermarkets, Singapore	μg/kg f.w.	32.8–164.5 Mean 72.5
Isobe et al. (2007)	Green mussel from India, Indonesia, Singapore, Malaysia, Thailand, Cambodia, Vietnam, and the Philippines during 1994–1999	μg/kg d.w.	1.1–13.7
Isobe et al. (2007)	Tokyo Bay	μg/kg d.w.	0.54–13.4
Shao et al. (2007)	Meat/seafood from supermarkets in Beijing, China	μg/kg f.w.	<0.30–7.08 Mean 0.71
Cao et al. (2011)	Different foods from stores in Quebec City, Canada	μg/kg f.w.	0.2–106 Mean 7.7
Noonan et al. (2011)	Canned food from local supermarkets in Washington and Maryland, USA	μg/kg f.w.	<2–790 Mean 509
Gyllenhammar et al. (2012)	Fruits, meats, and vegetables commercially available, Sweden	μg/kg f.w.	<2.0–29.0 Mean 3.8
Dodgen et al. (2013)	Lettuce and collards, steam and leaves	μg/kg f.w.	0.22–3.05
Dodgen et al. (2013)	Lettuce and collards, roots	μg/kg f.w.	199.6–441.7
Li et al. (2013c)	Soft commercial drinks	mg/m^3^	<0.02–0.86 Mean 0.31
Lu et al. (2013)	Vegetables and fruits in Florida, USA	μg/kg f.w.	0.2–9.0 Mean 4.2
Maggioni et al. (2013)	Drinking water from public drinking fountains, Italy	mg/m^3^	<0.00073–0.102
Maggioni et al. (2013)	Bottled mineral water, Italy	mg/m^3^	<0.00073–0.00113
Michałowicz (2014)	Meat products, worldwide	μg/kg f.w.	0.49–56
Michałowicz (2014)	Fish, worldwide	μg/kg f.w.	7.1–103
Michałowicz (2014)	Vegetables and fruits, worldwide	μg/kg f.w.	11–95
Michałowicz (2014)	Cereals, worldwide	μg/kg f.w.	1.0–3.8
Michałowicz (2014)	Various tinned products, including vegetables, fruits, and seafood, worldwide	μg/kg f.w.	0.1–267

#### BPA in soil and sediments

BPA concentrations in soils span between 0.55 and 147 μg/kg on dry weight basis (d.w.), with higher values generally found in agricultural fields amended with biosolids or irrigated with wastewater. Kinney et al. (2008) observed higher maximum concentrations of BPA in soils at a not-amended site (147 μg/kg d.w.) than at a site amended with biosolids (81 μg/kg d.w.); no detectable concentration of BPA was observed in site receiving liquid swine manure. Low values were observed by Xu et al. (2008) in soils from a golf course in southern California irrigated with reclaimed wastewater, but the authors pointed out the potential exposition of groundwater to contamination due to the accumulation of BPA over time. Gibson et al. (2010) investigated agricultural fields irrigated with wastewater for many years (up to 90 years) at different horizons; concentrations of BPA up to 30.2 μg/kg d.w. were measured, suggesting little evidence of BPA accumulation in soil and no evidence of transport through the different horizons.

Concentrations of BPA in sediments range between <0.24 and 492 μg/kg d.w. Values between 66 and 343 μg/kg d.w. were measured by Heemken et al. (2001) in the Elbe River; the highest concentration was observed downstream to a chemical factory. The authors monitored also the changes during 21 months of the BPA concentration in one of the most impacted sampling point, observing concentrations ranging between 127 and 322 μg/kg d.w. In Kawahata et al. (2004), the highest values were observed in areas affected by high pollution and/or downstream of commercial and industrial sites. Fu et al. (2007) studied the distribution of BPA in sediments collected in the Jiaozhou Bay (China) and at five monitoring stations located in the surrounding rivers; BPA was detected in all samples from the bay at concentrations between 0.7 and 20.3 μg/kg d.w. and at concentrations between 2.4 and 27.3 μg/kg d.w. in samples from the river sediments, resulting in similar ranges of concentrations. Pojana et al. (2007) investigated the occurrence of BPA in sediment samples collected at four stations in the Venice Lagoon close to municipal wastewater and industrial discharges; BPA was detected in seven out of eight samples at values up to 118 μg/kg d.w. in the sampling station nearest to the plant discharge.

#### BPA in groundwater and surface water

Reported concentrations of BPA in groundwater vary between 0.001 and 20 mg/m^3^. Lacorte et al. (2002) analyzed groundwater collected at an agriculture area of Catalonia (Spain) treated with a pesticide containing traces of BPA (1.5 mg/m^3^ in the pesticide), resulting in concentrations between <0.01 and 0.35 mg/m^3^. Latorre et al. (2003) performed measurements in groundwater collected from agriculture sites in northern Spain; the highest level (>1.5 mg/m^3^) was found next to grape cultivations. Godejohann et al. (2009) performed systematic analyses of groundwater near a former ammunition destruction site in Switzerland; BPA concentrations were about 12–13 mg/m^3^. In USEPA (2010a), the average concentrations of BPA in groundwater in the USA vary between 0.0041 and 1.9 mg/m^3^, with a range of values of 0.006–2.55 mg/m^3^. Stuart et al. (2011) reported about a survey on micropollutants in groundwater in England, with BPA concentrations up to 20 mg/m^3^; the distribution of detections was clearly split into two areas, southern England and Midlands.

BPA in surface water is reported between <0.001 and 92 mg/m^3^. Azevedo et al. (2001) detected BPA in 51 % of the analyzed samples of river and coastal waters from Portugal; authors observed values greater than 2 mg/m^3^ only in two sampling points located near industrial areas. Heemken et al. (2001) measured the concentration of BPA in the Elbe River and in some of its tributaries between 0.017 and 0.776 mg/m^3^; BPA probably originated from an industrial plant manufacturing epoxy resins at the German-Czech border and from a municipal sewage plant. Basheer et al. (2004) collected surface seawater samples at different locations along the Singapore coastline, encompassing both inshore and offshore sampling locations; BPA concentrations were between <0.002 and 2.47 mg/m^3^. In general, BPA concentration in samples obtained from offshore locations were lower than inshore samples. Kawahata et al. (2004) measured appreciable concentrations of BPA in water samples (between 0.036 and 0.08 mg/m^3^) in the most populated areas; values below detection limits (0.005 mg/m^3^) were measured at other locations. Vethaak et al. (2005) measured low concentrations of BPA (<0.009–1 mg/m^3^) in half of Dutch rainwater and surface water samples; the majority of samples had nanograms per liter, with few higher exceptions. Patrolecco et al. (2006) investigated the presence of BPA in water along the Tiber River (Italy); BPA concentrations were rather constant along the studied tract both in summer (0.06–0.09 mg/m^3^) and in winter (<0.03–0.14 mg/m^3^). Loos et al. (2007) monitored river waters in two textile industry regions, in Belgium (south Ghent) and in Italy (south Como). Similar values were obtained in both cases, with Belgian concentrations between <0.002 and 0.055 mg/m^3^ and Italian data between <0.002 and 0.175 mg/m^3^. Fu et al. (2007) measured BPA concentrations between 0.0015 and 0.0925 mg/m^3^ in water samples in the Jiaozhou Bay; higher values (an order of magnitude) were found in rivers surrounding the bay (0.0168–0.262 mg/m^3^). Pojana et al. (2007) measured BPA in water samples in the Venice Lagoon, with values between <0.001 and 0.145 mg/m^3^; the highest values were measured at the sampling stations in the proximity of an industrial effluent discharge point and of a municipal and industrial wastewater treatment plant. USEPA (2010a) reported a range of BPA average concentration in surface water of 0.012–0.14 mg/m^3^ and a range of data of 0.0009–12 mg/m^3^. Wu et al. (2013) measured concentrations of BPA in waters from Huangpu River and its tributaries (China) in winter and in summer, but the BPA levels in surface waters showed no clear seasonal pattern. Also Xu et al. (2014) did not observe seasonal variation of BPA in the seawaters of a marine reserve in Hong Kong. Gorga et al. (2015) carried out an extensive monitoring campaign on several Spanish rivers observing concentrations of BPA ranging from <0.00011 to 0.649 mg/m^3^; the higher values were found in well-known contaminated sites, near big cities or near industrial areas in the Llobregat and Ebro river basins.

#### BPA in biota and food

Many authors studied the migration of BPA and its derivatives from polymer packaging into food, especially under the effect of high temperature (e.g., microwave heating). Polycarbonate hydrolysis is the dominant mechanism responsible for BPA release from the polymer surface to the contacting liquids (Mercea 2009). Concentrations between 0.1 and 790 μg/kg fresh weight (f.w.) were found in food and <0.00073 and 0.86 mg/m^3^ in drinking waters/commercial drinks from different countries. The highest values in canned food were due to epoxy resins used as internal coating. For this reason, since 2001, epoxy resin films have been largely replaced with polyethylene terephthalate films (Huang et al. 2012; Wright-Walters et al. 2011). Basheer et al. (2004) measured concentrations of BPA in seafood samples (prawn, crab, blood cockle, white clam, squid, fish) purchased from a local supermarket in Singapore, resulting in significant values in all samples (between 13.3 and 213.1 μg/kg f.w.); the maximum value was found in crab. Sun et al. (2006) investigated canned food samples purchased in Singapore; detectable amounts of BPA were found in all samples, with concentrations between 32.8 and 164.5 μg/kg f.w. Shao et al. (2007) measured BPA in different types of fresh meat purchased in Beijing; BPA was detectable in 13 out of 27 samples, with concentrations between 0.33 and 7.08 μg/kg f.w. High concentrations of BPA were found in aquatic animals (fish and duck meat), due to the contamination of the aquatic environment. Cao et al. (2011) analyzed foods from four different stores in Quebec City (Canada); among the 154 food samples, BPA was detected in 55 samples, with concentrations from 0.20 to 106 μg/kg f.w. BPA levels in raw vegetable samples (not canned) were low, while BPA was detected at high concentrations in all the canned samples. BPA was not detected in any of the bottled water samples. Noonan et al. (2011) examined 78 canned and 2 frozen foods purchased from retail stores in Washington, DC, and in the surrounding Maryland (USA). BPA was detected in 71 out of 78 samples of canned foods, at concentration from <2 to 730 μg/kg f.w.; BPA was not detected in the frozen foods. The concentrations in canned fruits were lower than in other canned foods; data were consistent with the general industry practice of using tin and not epoxy phenolic films in canned fruit containers. Gyllenhammar et al. (2012) analyzed the levels of BPA in food from a typical food market basket purchased from two store chains in four major Swedish cities. Concentrations above the limit of quantification (2 μg/kg f.w.) were found in fish (2.5–29 μg/kg f.w.), meats (6.9–13 μg/kg f.w.), potatoes (2.2 μg/kg f.w.), and dairy products (2.4 μg/kg f.w.). Lu et al. (2013) analyzed BPA in fresh fruits and vegetables commercially available in Florida (USA); concentrations between 0.2 and 4.3 μg/kg f.w. and between 2 and 9 μg/kg f.w., respectively, were found. Dodgen et al. (2013) investigated the accumulation of BPA in different parts of lettuce and collards, pointing out the poor translocation of BPA from roots to the upper tissues after uptake: in the stem and leaves, concentration ranges 0.22–0.36 and 1.42–3.05 μg/kg f.w. were observed for lettuce and collards, respectively, but the values measured in the roots were greater by 3–2 orders of magnitude (441.7 and 199.6 μg/kg f.w.). Maggioni et al. (2013) evaluated concentrations of BPA in drinking waters from public fountains in 35 Italian cities and in bottled mineral waters; the highest concentration of BPA, 0.102 mg/m^3^, was detected only in one sample in water from public fountains, while in the other samples, the amount was only slightly above the quantification limit (0.00073 mg/m^3^).

Based on BPA concentrations in food and food consumption, a daily dietary BPA intake of 0.02–0.081 μg/kg/day for adults and 0.22–0.33 μg/kg/day for infants was estimated (Basheer et al. 2004; Sun et al. 2006; Shao et al. 2007; Cao et al. 2011; Gyllenhammar et al. 2012; Noonan et al. 2011; Lu et al. 2013). At present, the dietary intake of BPA appears to be the primary source for human exposure.

## Nonylphenol (NP)

NP is a term used to refer to a wide group of isomeric compounds (C_15_H_24_O) consisting of a nine-carbon alkyl chain bond to a phenol ring (Table 1). The various isomers can differ either in the degree of alkyl chain branching or in the position on the phenol ring. The NP isomers most produced and measured in the environment is 4-NP. NP is used as a formulant in pesticides, as a lubricating oil additive, as a catalyst in epoxy resins curing, at industrial laundries and, in the past, to produce nonylphenol ethoxylates (NPEs) for consumer products (e.g., surfactants, detergents, wetting agents, dispersants, defoamers, de-inkers, antistatic agents) (CCME 2002; European Parliament 2003; USEPA 2010b). As tris(4-NP)phosphite, it is an antioxidant for the stabilization of rubber, vinyl polymers, polyolefins, and styrenics. In 2010, the US demand for NP was 380 million pounds (ICIS 2007 in USEPA 2010b).

### Toxicity

NP is an estrogen agonist (ECHA 2014). It is highly irritating and corrosive to skin and eyes, but it does not have significant skin-sensitizing potential. The acute (oral and dermal) toxicity is low. NP carcinogenicity data give some reason for concern, though more data are needed (ICIS 2007). NP is highly toxic to fish, aquatic invertebrates, and aquatic plants (OEHHA 2009).

Bakke (2003) proposed a RfD value of 0.10 mg/kg/day, which should be protective for human health under chronic exposure to NP and NPEs, and the Danish Institute of Safety and Toxicology (DIST) derived a preliminary tolerable daily intake (TDI) value for NP of 5 μg/day/kg body weight (Danish EPA 2000).

### Environmental fate and transport

NP is a viscous liquid at room temperature (Kawahata et al. 2004; USEPA 2010b) and is primarily released into the environment through the discharge of municipal and industrial wastewater into surface waters (Writer et al. 2012). Pathways to the terrestrial environment include the spraying of pesticides containing NP or NPEs as formulates, landfilling of sludge, or the application of sewage sludge or pulp and paper mill sludge to agricultural soils (Soares et al. 2008; Brown et al. 2009). However, Brown et al. (2009) suggested that in practical field situations, where typical biosolids (NP <1000 mg/kg) are used at agronomic rates (<2.0 kg/m^2^), NP does not accumulate and plant uptake or water quality impairment is minimal.

NP can also be the products of biodegradation of alkylphenol polyethoxylates. These compounds, in fact, biodegrade by stepwise loss of ethoxy groups, resulting in the formation at the end of various hydrophobic alkylphenols (Ying and Kookana 2003). Thus, NP and NPEs are constituents of untreated and insufficiently treated wastewater and are also metabolites of widely used alkylphenolic compounds. It has been shown that the formation of NP is favored under anaerobic conditions, but it was also reported under aerobic conditions (Micic and Hofmann 2009). The formation of NP from its precursors has been observed in anaerobic sludge treatment (Ahel et al. 1994), surface reservoir sediments (Micić et al. 2013), and estuarine sediments (Lee Ferguson et al. 2003)

Due to its physical-chemical properties (Table 2), such as low water solubility and high log(*K*_OC_) values, sorption plays an important role on NP fate and transport in soil-water systems and river sediments (Bennie et al. 1997; Sekela et al. 1999; Azevedo et al. 2001; Bester et al. 2001; Heemken et al. 2001; Fries and Puttmann 2003; Jonkers et al. 2003; Rice et al. 2003; Basheer et al. 2004; Kawahata et al. 2004; Vitali et al. 2004; Cespedes et al. 2005; Vethaak et al. 2005; Patrolecco et al. 2006; Fu et al. 2007; Loos et al. 2007; Pojana et al. 2007; Wu et al. 2007; Micic and Hofmann 2009; Chen et al. 2013), where it is moderately persistent (OEHHA 2009; Li et al. 2013a). Liao et al. (2014) and Roberts et al. (2014) reported that the amount of soil organic matter dominated the sorption capacity of 4-NP and NP to different soils though a clear linear relationship was not evident. Sorption process reached equilibrium in 6 h, with a first rapid sorption stage (30 min ahead) followed by a slow sorption stage (30 min afterward).

Similar results were also reported for sorption in aquatic sediments in Ding et al. (2014), even slowing down of the process was observed in the presence of biofilm. Shchegolikhina et al. (2012), accordingly, observed also NP extractability from soil, with water and other agents, decreasing at increasing contact time with soil. In marine sediments, NP sorption is enhanced under high salinity (Yang et al. 2011).

NP is not likely to volatilize from soil and is rapidly degraded by hydroxyl radicals in the atmosphere (USEPA 2010b). NP is moderately bioaccumulative (OEHHA 2009).

NP undergoes photolysis in water. In Martínez-Zapata et al. (2013), it was degraded in ultrapure water due to direct photolysis under solar irradiation (300–800 nm) according to a first-order kinetic. Fe(III) and humic acids had a significant synergistic effect. Li et al. (2013b) investigated NP photolysis by sunlight. In pure water, the pseudo-first-order rate constant decreased from 6.73 × 10^−3^ to 1.57 × 10^−3^ l/min as the NP initial concentration increased from 40 mg/m^3^ to 5.0 g/m^3^; in seawater, the removal rate was slightly slower, the difference being ascribed to the presence of competing species. Similar results were observed by Neamtu and Frimmel (2006).

NP undergoes aerobic biodegradation in water, sediment, and soil systems, but high concentrations can be toxic to microorganisms (EC 2002). Mineralization has been observed in a variety of soil types, including agricultural soils of various textures, noncultivated temperate soils, and soils from the Canadian tundra (Topp and Starratt 2000 in CCME 2002). Gabriel et al. (2008) investigated the degradation of technical NP (a mixture of more than 100 isomers) by *Sphingobium xenophagum* Bayram; the strain degraded NP isomers differentially, being those with less bulkiness at the α-carbon and with four to six carbon atoms mainly alkyl chain being degraded more efficiently. Lu and Gan (2014) compared biodegradation kinetics of a large suite of NP isomers in river sediments under both oxic or anoxic conditions, reporting half-lives of NP isomers sediment ranging from 0.9 to 13.2 days under oxic conditions and from 15.1 to 20.1 days under slightly reduced conditions. Under reduced conditions, the persistence of NP isomers generally increased with estimated first-order half-lives of NP isomers greater than 200 days, with negligible dissipation under strongly reduced conditions.

Chang et al. (2004) observed anaerobic degradation of NP in a sediment-water system by sulfate-reducing bacteria, methanogens, and eubacteria. Fungi can degrade NP exclusively under aerobic conditions (Corvini et al. 2006a). Rozalska et al. 2010 tested filamentous fungi *Gliocephalotrichum simplex* to degrade 4-n-NP (50 g/m^3^), which was removed by 88 % after 24 h of incubation and almost completely after 48 h. In the same study, 4-n-NP at 100 g/m^3^ was also removed, but at a slower rate.

### Degradation byproducts

Li et al. (2013b) observed the formation of 4-nonyl-catechol after natural irradiation of NP in water. The authors also detected n-nonoic acid in irradiated pure water, but not in seawater.

Corvini et al. (2006b) studied the degradation pathways of NP by *Sphingomonas* sp. TTNP3, a microbial strain that exhibited high degradation capabilities toward NP used as sole carbon and energy source. The major metabolite in the degradation pathway was hydroquinone, which was further degraded to organic acids (succinate and 3,4-dihydroxy butanedioic acid); benzenediol and alkyloxy derivatives were the dead-end products. Rozalska et al. (2010) investigated the metabolic degradation pathway of 4-n-NP by the nonligninolytic filamentous fungi *G. simplex*, resulting in two possible routes. In one route, carbon detachment brought to 4-hydroxyphenylheptanoic acid and then to 3-(4-hydroxyphenyl)propanoic acid, which was transformed into 4-(1-hydroxyvinyl)phenol, on a side route, and to 2-(4-hydroxyphenyl)acetic acid and 4-hydroxybenzoic acid, on the main route. In the second route, hydroxylation at the ninth position (close to the aromatic ring) and carboxylation at the first position (distal carbon) of the nonyl-moiety brought to 9-hydroxy-9-(4-hydroxyphenyl)nonanoic acid. In 6 h of incubation, 4-hydroxybenzoic acid was the major metabolite. After 72 h of incubation, no toxic effects were observed.

### Environmental evidence

Table 4 summarizes the major studies on NP concentrations in different environmental matrices and food.

**Table 4:** NP concentrations in various environmental matrices and in food (percentages between brackets represent the detection frequency)

Reference	Location	Units	Value
Soils			
CCME (2002)	Soil amended with sludge, Canada	μg/kg d.w.	2720
Vikelsøe et al. (2002)	Unamended, manured or artificially fertilized soils and soils amended with limited amounts of sewage sludge, Denmark	μg/kg d.w.	0.01–0.98 Mean 0.37
	Soil amended with high amounts of sewage sludge, Denmark	μg/kg d.w.	1450–2430 Mean 1940
Gibson et al. (2010)	Agricultural fields irrigated with wastewater, Tula Valley, Mexico	μg/kg d.w.	<25–299
Sediments			
Bennett and Metcalfe (1997)	Great Lakes, USA and Canada	μg/kg d.w.	<46–37,800 Mean 3000
Bennie et al. (1997)	Great Lakes and St. Lawrence River in 1995, USA and Canada	μg/kg d.w.	170–72,000 Mean 10,600
Yamashita et al. (2000)	Tokyo Bay, Japan	μg/kg d.w.	<10–5540
Bester et al. (2001)	Bight in the North Sea, Germany	μg/kg d.w.	10–153 Mean 55
	Open sea, North Sea, Germany	μg/kg d.w.	<10–55 Mean 34 (40 %)
Heemken et al. (2001)	Elbe River and some of its tributaries, Germany	μg/kg d.w.	367–1378 Mean 640
Jonkers et al. (2003)	Western Scheldt and Rhine estuaries, Holland	μg/kg d.w.	<0.4–1080 Mean 19.5 (94 %)
Kannan et al. (2003)	Kalamazoo River, USA	μg/kg d.w.	<5.5–15.3
Rice et al. (2003)	Cuyahoga River, Ohio (USA)	μg/kg d.w.	75–340 Mean 180
Kawahata et al. (2004)	Estuarine and marine sediments from Okinawa, and Ishigaki Islands, Japan	μg/kg d.w.	<5–46 Mean 30.5 (47 %)
Vitali et al. (2004)	Rieti District, Italy	μg/kg d.w.	44–567 Mean 205
Vethaak et al. (2005)	Fresh, marine, and estuarine sediments, The Netherlands	μg/kg d.w.	<10–3800 Median 160 (91 %)
Lara-Martin et al. (2006)	Marine and estuarine sediments from Bay of Cadiz, Spain	μg/kg d.w.	13–225 Mean 108
Patrolecco et al. (2006)	Tiber River, Italy	μg/kg d.w.	50–970 Mean 414
Fu et al. (2007)	Estuarine and marine sediments from Jiaozhou Bay and surrounding rivers, China	μg/kg d.w.	3.6–39,700 Mean 3670
Pojana et al. (2007)	Sediments in Venice Lagoon, Italy	μg/kg d.w.	47–192 Mean 89
Wu et al. (2007)	Urban lakes in Wuhan City, China	μg/kg d.w.	3540–32,430 Mean 10,490
Micic and Hofmann (2009)	Danube River, Germany	μg/kg d.w.	<20–2830 Mean 130
Gong et al. (2011)	Major tributaries in Pearl River system, China	μg/kg d.w.	31–21,885 Mean 3686
Klosterhaus et al. (2013)	San Francisco Bay, USA	μg/kg d.w.	21.5–86.3 Mean 34.7
Micić et al. (2013)	Iron Gate I Reservoir on the Danube River, Romania	μg/kg d.w.	80–470
Wu et al. (2013)	Huangpu River and its tributaries, China	μg/kg d.w.	10.34–337.73
Koniecko et al. (2014)	Surface sediments of the Gulf of Gdansk, Poland—rivers	μg/kg d.w.	<0.08–4.93
	Surface sediments of the Gulf of Gdansk, Poland—coastal stations	μg/kg d.w.	<0.08–13.56
	Surface sediments of the Gulf of Gdansk, Poland—stations below 4 m depth	μg/kg d.w.	<0.08–249.08
Duan et al. (2014)	Surface sediments of the Yellow Sea and East China Sea, China	μg/kg d.w.	349.5–1642.8 Mean 890.1
Gorga et al. (2014)	Ebro River basin, Spain	μg/kg d.w.	36–538 Mean 177
Stewart et al. (2014)	Estuarine sediments in Auckland, New Zealand	μg/kg d.w.	<100–32,000 Median 153 4-n-NP <100
Gorga et al. (2015)	Sediments from Ebro, Llobregat, Júcar and Guadalquivir rivers, Spain	μg/kg d.w.	<0.24–1693
Groundwater			
Lacorte et al. (2002)	Agricultural area in Catalonia, Spain	mg/m^3^	<0.01–0.35
Latorre et al. (2003)	Agricultural areas in northern Spain	mg/m^3^	<0.036–0.9 (92 %)
Félix-Cañedo et al. (2013)	Groundwater in Mexico City, Mexico	mg/m^3^	<0.001–0.047 (43 %)
Loos et al. (2010), Luo et al. (2014)	23 European countries, Europe	mg/m^3^	<0.030–3.85 Mean 0.083 (11 %) 90th percentile 0.039
Surface Water			
Bennie et al. (1997)	Great Lakes and St. Lawrence River in 1995, USA and Canada	mg/m^3^	<0.01–0.92 Mean 0.21 (24 %)
Sekela et al. (1999)	Upstream of a WWTP, Fraser River, Canada	mg/m^3^	0.0066–0.0074
	Downstream of a WWTP, Fraser River, Canada	mg/m^3^	0.032–0.13
Azevedo et al. (2001)	River and coastal waters, Portugal	mg/m^3^	<0.01–30 Mean 1.2 (79 %)
Bester et al. (2001)	Bight of the North Sea, Germany	mg/m^3^	0.0007–0.033
Heemken et al. (2001)	Elbe River and its tributaries, Germany	mg/m^3^	0.0008–0.221 Mean 0.059
	North Sea	mg/m^3^	0.0003–0.084 Mean
Fries and Puttmann (2003)	Rhine, Elbe, Main, Oder, Nidda, and Schwarzbach rivers, Germany	mg/m^3^	<0.025–1.22 Mean 0.43
Jonkers et al. (2003)	Western Scheldt and Rhine estuaries, Holland	mg/m^3^	0.031–0.934 Mean 0.17
Kannan et al. (2003)	Kalamazoo River, USA	mg/m^3^	<2.6
Rice et al. (2003)	Cuyahoga River, Ohio (USA)	mg/m^3^	0.1–0.5 Mean 0.24
Basheer et al. (2004)	Surface coastal water, Singapore	mg/m^3^	0.02–2.76 Mean 0.95
Kawahata et al. (2004)	Estuarine and marine waters from Okinawa, and Ishigaki Islands, Japan	mg/m^3^	<0.05–0.17 Mean 0.14 (29 %)
Vitali et al. (2004)	Rieti District, Italy	mg/m^3^	<0.1–1.6
Cespedes et al. (2005)	Llobregat River basin, Catalonia, Spain	mg/m^3^	<0.15–37.3 Mean 5.7 (90 %)
Vethaak et al. (2005)	The Netherlands	mg/m^3^	<0.11–4.1 Median 0.99 (10 %)
Patrolecco et al. (2006)	Tiber River, Italy	mg/m^3^	0.13–0.58 Mean 0.28
Vousta et al. (2006)	Glatt river, Switzerland	mg/m^3^	0.068–0.326
Fu et al. (2007)	Marine water from the Jiaozhou Bay, China	mg/m^3^	0.02–0.269
	Jiaozhou Bay inflowing rivers, China	mg/m^3^	0.0906–28.6
Loos et al. (2007)	River water, Belgium	mg/m^3^	0.32–2.50 Mean 1.44
Loos et al. (2007)	River water, Italy	mg/m^3^	0.46–0.70 Mean 0.56
Pojana et al. (2007)	Venice Lagoon, Italy	mg/m^3^	<0.0005–0.21
Wu et al. (2007)	Urban lakes in Wuhan City, China	mg/m^3^	1.94–32.85 Mean 11.96
Micic and Hofmann (2009)	Danube River, Germany	mg/m^3^	<0.1–0.13 (18 %)
Félix-Cañedo et al. (2013)	Surface water (dams) in Mexico City, Mexico	mg/m^3^	<0.001–0.655 (75 %)
Klosterhaus et al. (2013)	San Francisco Bay, USA	mg/m^3^	<0.00252–0.0729 (60 %)
Wu et al. (2013)	Huangpu River and its tributaries, China—July 2010	mg/m^3^	0.0202–0.1075 Mean 0.074
	Huangpu River and its tributaries, China—November 2010	mg/m^3^	0.0926–0.3317 Mean 0.1606
Esteban et al. (2014)	Manzanares and Jarama rivers, Spain	mg/m^3^	0.096–1.483
Luo et al. (2014)	China	mg/m^3^	0.036–33.231
	Greece	mg/m^3^	0.558–2.704
	Korea	mg/m^3^	0.115–0.336
Xu et al. (2014)	Seawater Cape D’Aguilar Marine Reserve, Hong Kong—wet season	mg/m^3^	0.14–0.50 Mean 0.39
	Seawater Cape D’Aguilar Marine Reserve, Hong Kong—dry season	mg/m^3^	0.061–0.33 Mean 0.11
Zhang et al. (2014)	North Tai Lake Basin, Eastern China	mg/m^3^	0.089–1.189 Mean 0.388
Gorga et al. (2015)	Iber Ebro, Llobregat, Júcar, and Guadalquivir rivers (Ebro, Llobregat, Júcar, and Guadalquivir)	mg/m^3^	<0.00013–0.391
Food and biota			
Guenther et al. (2002)	Packed foodstuff from supermarkets, Germany	μg/kg f.w.	0.1–19.4 Mean 6.0
Rice et al. (2003)	Carps from Cuyahoga River, Ohio (USA)	μg/kg f.w.	6.6–110 Mean 53.4
Basheer et al. (2004)	Seafood from supermarkets, Singapore	μg/kg f.w.	46.6–197 Mean 87.7
Loyo-Rosales et al. (2004)	Spring water bottled in HDPE and PVC from supermarkets, USA	mg/m^3^	0.015–0.300 Mean 0.104
Ferrara et al. (2005)	Edible marine species from Adriatic Sea, Italy	μg/kg f.w.	2.7–1286 Mean 413
Vethaak et al. (2005)	Edible freshwater specie (bream), The Netherlands	μg/kg f.w.	<10–160 Median 135 (24 %)
Vethaak et al. (2005)	Edible marine specie (flounder) from North Sea Canal, The Netherlands	μg/kg f.w.	<10–10 Median 10 (10 %)
Isobe et al. (2007)	Green mussel from India, Indonesia, Singapore, Malaysia, Thailand, Cambodia, Vietnam, and the Philippines during 1994–1999	μg/kg d.w.	18–663 (79 %)
	Tokyo Bay during 1994–1999	μg/kg d.w.	47–1347
Shao et al. (2007)	Meat/seafood from supermarkets in Beijing, China	μg/kg f.w.	<0.05–55.98 Mean 6.87
Ferrara et al. (2008)	Edible marine species from Tyrrhenian Sea, Italy	μg/kg f.w.	5–1220 Mean 147
Cacho et al. (2012)	Plastic packed vegetables from local supermarkets, Spain	μg/kg f.w.	<14.5–48 (14 %)
Diehl et al. (2012)	Marine organisms California estuary, Morro Bay, USA	μg/kg f.w.	122–2380
Gyllenhammar et al. (2012)	Fruits, cereal products, and vegetables commercially available, Sweden	μg/kg f.w.	<10–71
Dodgen et al. (2013)	Lettuce and collards, steam and leaves	μg/kg f.w.	1.18–6.95
Dodgen et al. (2013)	Lettuce and collards, roots	μg/kg f.w.	339.2–926.9
Li et al. (2013c)	Soft commercial drinks	mg/m^3^	<0.03–0.22 (25 %)
Lu et al. (2013)	Vegetables and fruits, from local commercial sources, Florida (USA)	μg/kg f.w.	<0.3–11.0 4-n-NP <0.1–18.5
Maggioni et al. 2013	PET - bottled water	mg/m^3^	<0.0077
Maggioni et al. (2013)	Drinking water from public drinking fountains, Italy	mg/m^3^	<0.0077–0.084 (23 %)
Dodder et al. (2014)	Mussels along the California coast, USA	μg/kg d.w.	96–3000 Mean 470 Median 200

#### NP in soil and sediments

Few studies are available in the literature on NP occurrence in soil. All the authors focused on agricultural soils to highlight the effects of sludge amendment and irrigation with untreated wastewater.Vikelsøe et al. (2002) studied the distribution of NP in dressed and fertilized agricultural soils in Denmark. NP concentrations between 0.01 and 0.98 μg/kg d.w. were found in unamended soils, soils fertilized with manure, or with limited amounts of sewage sludge and 34 μg/kg at runoff points. Higher concentrations of NP (1.45–2.43 mg/kg), persisting up to 8 years after amendment had ceased, were instead measured in soils exposed to a high addition of sewage sludge. Similar NP values were also reported for sludge-amended site in Canada (CCME 2002). In Mexican agriculture fields irrigated with untreated wastewater for 10 to 90 years, Gibson et al. (2010) measured NP ranging between <25 and 299 μg/kg d.w., indicating only little evidence of NP accumulation in soils and poor evidence of migration through the different horizons in soil.

Analytical data of sediment samples point out a variability up to 4 orders of magnitude: the reported concentrations of NP range from 3.6 μg/kg d.w. to 72 mg/kg d.w., with the highest values registered in lakes (Bennett and Metcalfe 1997; Wu et al. 2007). In sediments, high NP concentrations were generally associated to specific point sources such as industrial plants, or large amount of domestic wastewater entering the river as it flows through urban areas, especially in most populous regions (Fu et al. 2007; Wu et al. 2007; Duan et al. 2014; Stewart et al. 2014). Typically increasing from upstream to downstream was also reported, with higher values often registered in estuarine and coastal sediments (Fu et al. 2007; Gong et al. 2011; Gorga et al. 2015). The presence of NP in freshwater sediments was primarily ascribed to domestic and industrial wastes and to a lesser extent by agricultural activities (pesticide applications, sludge amendment, and irrigation with wastewater). Koniecko et al. (2014) recognized the rivers and surface runoff as the main sources of NP in coastal sediments of the Gulf of Gdansk; however, they also indicated the possibility of atmospheric transportation of black carbon originating from combustion processes on land along with adsorbed alkylphenols. In Central Europe, Micic and Hofmann (2009) detected concentrations of NP between <0.02 and 2.83 mg/kg d.w. in sediment samples collected along the Danube River; important point sources were industrial sites, especially oil refineries, and drains of untreated wastewater. Similar ranges were also measured along the Elbe River and some of its tributaries in Germany (Heemken et al. 2001) and for Rhine and the Western Scheldt estuaries in heavily industrialized areas or in areas receiving both treated and untreated domestic wastewater in the Netherlands (Jonkers et al. 2003). In Italy, concentrations of NP were measured in areas with different soil uses (urban, industrial, agricultural, open country) in the Rieti District, along the Tiber River and in the Venice Lagoon with similar values, ranging between 44 and 970 μg/kg d.w. (Vitali et al. 2004; Patrolecco et al. 2006; Pojana et al. 2007).

In marine sediments, a decrease in NP concentrations with respect to the levels measured in estuarine sediments and also with increasing distances from the coast was often observed (Bester et al. 2001; Vethaak et al. 2005). As an example, Fu et al. (2007) reported NP concentrations between 3.6 and 299 μg/kg d.w. in the sediments of Jiaozhou Bay (China) and between 23.8 and 39700 μg/kg d.w. in sediments of inflowing rivers.

In open-sea sediments, some offshore oil/gas drilling platforms were identified as a likely source of NPEs and NP, even if it is not clear if these are due to drilling and production activities or discharges from ships (e.g., cleaning activities) in the areas (Vethaak et al. 2005). Similar to non- and slightly polar organics, a positive correlation of NP with organic carbon content of the sediments was reported, confirming a role of organic carbon for sorption (Jonkers et al. 2003; Gorga et al. 2015). For NP concentrations in sediment, a clear seasonal pattern was not reported as samples may be deposited during a long time.

#### NP in groundwater and surface water

NP was measured in groundwater by Lacorte et al. (2002) and Latorre et al. (2003) in two agricultural areas of Spain, with values between below 0.01 (detection limit) and 0.9 mg/m^3^. More recent monitoring of groundwater resulted in measured NP concentration ranging between the limit of detection, 0.001 mg/m^3^, and 3.85 mg/m^3^ (Loos et al. 2010; Félix-Cañedo et al. 2013; Luo et al. 2014). NP contamination of groundwater was mainly associated to landfill leachate, water from agricultural land, or seepage of septic tanks and sewer systems (Luo et al. 2014).

The measured concentrations of NP in surface waters span between 3 × 10^−4^ and 37.3 mg/m^3^. Considering NP is highly hydrophobic and liable to adsorb to suspended solids and eventually to accumulate in sediments, authors comparing concentrations of sediments and surface water samples from the same location often observed higher concentration in surface sediments than in surface water (Wu et al. 2007, 2013; Micić et al. 2013). Vitali et al. (2004) also pointed out that recorded dissolved NP high levels were limited to a short distance (a few kilometers) downstream from the source of contamination, as due to chemical-physical characteristics and their adsorption on particulate matter, sediments represent the final sink for NP. As a consequence, high NP in sediments and low concentrations in water was often ascribed to past emissions (Jonkers et al. 2003).

Nevertheless, the spatial distribution of NP in surface water was quite the same of the NP concentrations in sediments, with the highest levels closely related to the input of industrial or domestic wastewater discharges or wastewater treatment plant effluents (Bennie et al. 1997; Sekela et al. 1999; Kannan et al. 2003; Cespedes et al. 2005) and a general increase from the upper rural portions of the river to the urbanized and industrialized segments (Esteban et al. 2014; Luo et al. 2014). Azevedo et al. (2001) in Portugal registered especially high (up to 30 mg/m^3^) NP concentrations in industrial districts where tannery and textile industries are located. Loos et al. (2007), in Belgium (south Ghent) and in Italy (south Como), analyzed wastewater treatment plant (WWTP) effluents of textile industries and the receiving rivers and found NP concentrations in the receiving waters upstream the effluent discharge (0.32–2.50 mg/m^3^) comparable to NP levels in WWTP effluents (0.37–0.73 mg/m^3^). Despite agriculture is recognized as a minor source of NP in freshwater, Patrolecco et al. (2006) measured NP above 0.30 mg/m^3^ in water samples collected from the Tiber River (Italy) at heavily exploited rural areas.

In freshwaters in Central Europe, NP up to about 1.3 mg/m^3^ has been reported (Jonkers et al. 2003; Fries and Puttmann 2003; Micic and Hofmann 2009; Vethaak et al. 2005). By comparing the results of past water monitoring with more recent data, it was also highlighted a decrease in dissolved NP concentrations in European rivers in the last two decades, probably as consequence of the various voluntary restrictions or legislation on the use of NP in household cleaning products and industrial applications together with possible relocation of industrial activities (Fries and Puttmann 2003; Vousta et al. 2006; Micic and Hofmann 2009; Gorga et al. 2014).

In coastal areas, a significant decreasing trend in NP concentrations with the distance from the coast is often reported (Basheer et al. 2004; Heemken et al. 2001; Fu et al. 2007). Significant levels of NP have been occasionally reported in offshore sampling points in the vicinity of industrial areas, jetties, shipyards, marinas and recreational beaches, as well as in shipping lanes, anchorages, and near petroleum refineries (Basheer et al. 2004).

Fu et al. (2007) and Xu et al. (2008) reported of a seasonal trend of dissolved NP concentrations with higher values in summer than in winter. Such finding was ascribed firstly to high temperatures and associated microbial activity, leading to an enhanced degradation of NPEs in marine sediments and hence an increased NP concentrations in water column during summer. In the Hong Kong area, as the summer is also the wet season, it was also assumed a possible effect on NP level in water due to increased surface runoff during rain events (Xu et al. 2008). Kueh and Lam (2008) in Hong Kong measured in storm water NP concentrations between 0.08 and 12 mg/m^3^.

#### NP in biota, food, and bottle water

Significant NP concentrations were found in different foods, with values between 0.1 and 100 μg/kg f.w. and <7.7 μg/m^3^ and 0.30 mg/m^3^ in drinking waters/commercial drinks from different countries. In Guenther et al. (2002), NP concentrations in packed foodstuff purchased from supermarkets in Germany spanned between 0.1 and 19.4 μg/kg f.w. The concentration was not related to the food fat content and NP migration into food occurred at different stages of the food production.

High NP concentrations in seafood and various edible marine species were observed in Asia (Basheer et al. 2004; Isobe et al. 2007; Shao et al. 2007), Europe (Ferrara et al. 2005, 2008), and North America (Dodder et al. 2014) at comparable levels, considering the differences in species examined in each study, number of collected samples, period, analytical methods and reporting units, and also different periods.

Measured values of NP in different commercially available vegetables and fruits in Sweden (Gyllenhammar et al. 2012), Spain (Cacho et al. 2012), and Florida (Lu et al. 2013) varied roughly between 5 and 50 μg/kg f.w.

Different accumulation of NP in each species was observed. Significant values were found in carrots and pumpkins (10.4 and 11.3 μg/kg f.w., respectively) and in apples and citruses (17.1 and 29.5 μg/kg f.w., respectively), whereas NP was not detected in strawberries, lettuce, potato, and tomatoes. Dodgen et al. (2013) investigated the accumulation of NP in different parts of lettuce and collards, pointing out the poor translocation of NP from the roots to the upper tissues after uptake: in the stem and leaves, concentration ranges 1.18–4.31 and 3.79–6.95 μg/kg f.w. were observed for lettuce and collards, respectively, but the values measured in the roots were greater by 3 orders of magnitude (926.9 and 339.2 μg/kg f.w. for lettuce and collards, respectively).

Loyo-Rosales et al. (2004) investigated the presence of NP in commercial water bottled in different materials (high-density polyethylene (HDPE), polyethylene terephthalate (PET), polyvinyl chloride (PVC)). NP was found in water contained in HDPE and PVC bottles at values of 29–180 and 15–300 μg/m^3^, respectively. Maggioni et al. (2013) evaluated concentrations of NP in drinking waters from public fountains in 35 Italian cities and in bottled mineral waters; in all samples, the amount varied from below the quantification limit (7.7 μg/m^3^) up to a maximum of 84 μg/m^3^. These values are similar to the reported NP range in commercial soft drinks (Li et al. 2013c).

Based on the concentrations measured in food and the expected consumption rates, the average daily intake of NP varies between 0.067 and 0.370 μg/kg/day for adults (60 kg body weight) (Guenther et al. 2002; Ferrara et al. 2005, 2008; Shao et al. 2007; Gyllenhammar et al. 2012; Lu et al. 2013). Loyo-Rosales et al. (2004) calculated an average NP daily intake from drinking bottled water of 0.36–0.60 μg/day. Diet seems the major exposure route for humans.

## Benzophenones (BPs)

Benzophenone (diphenyl ketone, BP, (C_6_H_5_)_2_CO) is composed of two aromatic rings and a carbonyl group (Table 1) (NTP 2006). Based on this parental structure, a group of different compounds can be generated through substitutions of hydrogen atoms in the aromatic rings. The physical-chemical properties and the environmental behavior of these derived compounds are not significantly different from those of the parent compound, except for benzophenone-3 (2-hydroxy-4-methoxybenzophenone, BP-3, Table 1), which is also the most commercialized compound of the group (León et al. 2010; Gago-Ferrero et al. 2012; Liu et al. 2012a; Zhang et al. 2013b).

BPs are used as a flavor ingredient, a fragrance enhancer, a perfume fixative, and an additive for plastics, coatings, and adhesive formulations. They are also used in laundry and household cleaning products and in the manufacture of insecticides, agricultural chemicals, hypnotic drugs, antihistamines, and other pharmaceuticals. BPs are used as an ultraviolet (UV)-curing agent in sunglasses and to prevent UV light from damaging scents and colors in products such as perfumes and soaps. BP-3 is commonly used worldwide as a UV filter in cosmetic formulations, such as sunscreens and skin care products, body lotions, hair sprays, hair dyes, and shampoos (Zhang et al. 2011; Liu et al. 2012a). Moreover, they can be added to plastic packaging as a UV blocker, which allows manufacturers to package their products in clear glass or plastic rather than opaque or dark packaging. BPs are widely used as a photoinitiator for inks and varnishes that are cured with UV light (Ricking et al. 2003; Zhang et al. 2011). In 2003, BP production exceeded 453 t in the USA and 10,000 t in the European Union (NTP 2006).

### Toxicity

BPs have adverse effects on reproduction and hormonal functions of fish (IARC 2010). They can alter endocrine signaling through multiple effects on receptors. The estrogenic activity of BP-3 and BP-1 (2,4-dihydroxybenzophenone) was determined by the estrogenic recombinant yeast assay, resulting in the half-maximal effective concentrations (EC_50_) of 12.5 and 0.058 g/m^3^, respectively, and a lowest observed effect concentrations (LOEC) of 1.6 and 0.015 g/m^3^, respectively (Gago-Ferrero et al. 2012). No data are available on BP carcinogenicity to humans, though they are classified as group 2B substances, “possible carcinogenic to humans” (NTP 2006; IARC 2010).

Because of their use as an additive in fragrances, cosmetics, pharmaceuticals, insecticides, and household cleaning products, exposure to BPs through dermal contact may be significant. In León et al. (2010), BP-3 applied on the skin was absorbed and readily biotransformed into BP-1, 2,2′-dihydroxy-4-methoxybenzophenone (BP-8), and 2,3,4-trihydroxybenzophenone (THB), whose decrease over time was much slower than the parent compound. Dietary sources of exposure include food and drinking water, where BPs might be present due to the addition as a flavoring or the migration from packaging (IARC 2010). The European Commission Scientific Committee on Food set a RfD for oral exposure of 10 μg/kg/day (EC 2005).

### Environmental fate and transport

BPs can enter the environment through solid-waste landfill leachate and wastewater treatment plants effluents (Ricking et al. 2003; Jeon et al. 2006). BP is insoluble in water (Table 2). Because of the high *K*_OC_ value, BP sorption on soil and sediment organic matter is significant (USEPA 1984). Volatilization can occur to some extent, but due to low vapor pressure, BP is not expected at significant levels in ambient air (USEPA 1984). BP in water can be photodegraded under sunlight exposure (Hayashi et al. 2006). Fujii and Kituchi (2005) observed BP biodegradation in activated sludge caused by a specific microbial strain. BP is persistent in the environment and susceptible to bioaccumulation (Brooks et al. 2009; IARC 2010). Based on the estimated *K*_OC_ value (Table 2), BP-3 has slight mobility in soil and sorbs significantly on suspended solids and sediments. Volatilization from soil surface and water is not expected to be an important process, though BP-3 in ambient air can exist in both vapor and particulate phase. The potential for BP-3 bioconcentration in aquatic organisms is moderate to high (TOXNET 2014).

BP-3 absorbs light at 288 and 326 nm and, therefore, is susceptible to direct photolysis by sunlight. However, Rodil et al. (2009) assessed the photostability of BP-3 under sunlight exposure (290 to 800 nm) of water samples spiked at 100–4000 mg/m^3^, without any significant decrease of BP-3 concentration over a 72 h irradiation period. Also, Gago-Ferrero et al. (2012) did not observe photodegradation of BP-3 over a 24 h irradiation period. Vapor-phase BP-3 is degraded in the atmosphere by reaction with photochemically produced hydroxyl radicals, with an estimated half-life of 1.9 h.

BP-3 can be biodegraded in water and soil systems. Liu et al. (2011a) and Liu et al. (2012a) investigated BP-3 biodegradation under oxic and anoxic conditions in water, resulting in BP-3 complete removal in both kinds of microcosms after 42 days of incubation. Anoxic conditions were more favorable than oxic conditions, with measured half-lives of 4.2 and 10.7 days, respectively. Gago-Ferrero et al. (2012) observed high biodegradation rates for BP-3 by white rot fungi *Trametes versicolor*, down to nondetectable levels in 8 h of incubation.

### Degradation byproducts

Hayashi et al. (2006) investigated BP byproducts after exposure of an aqueous solution to UV or sunlight irradiation; two-ring hydroxylated derivatives were observed (3-hydroxybenzophenone and 4-hydroxybenzophenone, 4HB).

According to Liu et al. (2011a) and Liu et al. (2012a), after 42 days of incubation in microcosms, biodegradation of BP-3 under Fe(III)-reducing conditions produced 4-cresol and BP-1, while BP-1 was detected under oxic, nitrate-reducing and sulfate-reducing conditions.

BP-1 was identified by Gago-Ferrero et al. (2012) as a metabolite produced during degradation of BP-3 by *T. versicolor*. BP-1 was then degraded to 4,4′-dihydroxybenzophenone (4DHB) and 4HB. More investigation is necessary to identify transformation products formed in environmental matrices in order to assess the potential environmental risk for BPs compounds (Jurado et al. 2014).

### Environmental evidence

The major papers in the literature reporting of BPs in environmental matrices and food are summarized in Table 5. Aquatic ecosystems in highly urbanized areas are the most important pathway for BPs to enter in the environmental matrices. Shortcomings in wastewater treatment  were reported with a removal efficiency for BP-3 ranging between 55–96 % (Kim and Choi 2014); recently, Ávila et al. (2014) observed a value equal to 93 %. Oxygen availability promote CEC degradation via aerobic pathways (example for BPA and BP-3); however, the releases of BP products are high due to human and industry activity (Kasprzyk-Hordern et al. 2008). Recently, for BP-3, the most ubiquitous compound, some authors paid attention to the direct diffusion due to the release of sunscreen products in water (Sánchez-Brunete et al. 2011; Kim and Choi 2014). In fact, maximum levels of contamination were observed in swimming pool or, in summer, during bath in recreational areas. In addition to the mentioned compounds (BP, BP-3, BP-1, 4HB, 4DHB, BP-8, THB), in the matrices, benzophenone-4 (BP-4), 2,2-dihydroxy-4.4-dimethoxybenzophenone (BP-6), and benzhydrol (BH) were also found.

**Table 5:** BP concentrations in various environmental matrices and in food (percentages between brackets represent the detection frequency)

Reference	Compound and location	Units	Value
Soils			
Jeon et al. (2006)	BPs, South Korea	μg/kg d.w.	BP 0.82–16.55, mean 4.55 (97 %) BP-3 0.73–3.88, mean 2.65 (15 %) BP-1 <0.5 BP-6 0.5–4.17, mean 1.67 (15 %) BH 0.51–6.95, mean 1.8 (39 %) 4HB 1.06–4.91, Mean 3.01 (9 %) THB <0.5
Sánchez-Brunete et al. (2011)	BPs, industrial, and agricultural areas, Spain	μg/kg d.w.	BP-1 <0.1–5.7 (ind) BP-6 <0.09–0.6 (agr) BP-3 <0.1 BP-8 <0.07 4HB <0.07
Sediments			
Jeon et al. (2006)	BPs, South Korea	μg/kg d.w.	BP 1.52–9.73, mean 4.73 (93 %) BP-3 <0.1 BP-1 <0.1 BP-6 0.5–2.14, mean 0.95 (80 %) BH 0.53, mean 0.53 (7 %) 4HB 18.38, mean 18.38 (7 %) THB <0.1
Pojana et al. (2007)	BP, Venice Lagoon, Italy	μg/kg d.w.	14–110 Mean 39.4
Sánchez-Brunete et al. (2011)	BPs, river and coastal sediments, Spain	μg/kg d.w.	BP-6 <0.15–6.1, mean 1.6 BP-1 <0.21 BP-3 <0.28 BP-8 <0.14 4HB <0.23
Kameda et al. (2011)	BP, rivers and lakes, Saitama Prefecture, Japan	μg/kg d.w.	2.7–105 Mean 34.7
Zhang et al. (2011)	BP-3, Songhua River, China	μg/kg d.w.	BP-3 0.272–0.545, mean 0.380 (100 %) BP-1 <0.14 BP-6 <0.22 4HB <0.22
Zhang et al. (2011)	BPs, Saginaw and Detroit River, USA	μg/kg d.w.	BP-3 0.728–4.66, mean 2.34 (100 %) BP-1 0.259–0.607, mean 0.454 (67 %) BP-6 0.133–0.796, mean 0.424 (67 %) 4HB 0.312–0.951, mean 0.53 (50 %)
Barón et al. (2013)	BP-3, river areas, estuary and coastal bays, Biobio region, Chile	μg/kg d.w.	<0.4–2.96
Barón et al. (2013)	BP-3, Magdalena River, Colombia	μg/kg d.w.	<0.4–5.38
Kim and Choi (2014)	BP-3, rivers, worldwide	μg/kg d.w.	<0.5–27
Groundwater			
Stuart et al. (2011)	BP, England	μg/m^3^	<10–2780
Jurado et al. (2014)	BPs, Barcelona urban groundwater	μg/m^3^	BP-1 mean 0.9 (16 %), max 19.4 BP-3 mean 2.3 (32 %), max 19.2 BP-4 mean 2.8 (19 %), max 36.6 4HB mean 0.2 (6 %), max 3.5 4DHB mean 0.13 (6 %), max 4.1
Jurado et al. (2014)	BPs, Mallorca street zone	μg/m^3^	BP-1 mean 0.78 (43 %), max 3.2 BP-3 mean 7.9 (71 %), max 19.2 BP-4 mean 1.1 (25 %), max 6.4 4HB mean 0.38 (14 %), max 2.6 4DHB mean 0.58 (14 %), max 4.1
Jurado et al. (2014)	BPs, Poble Sec zone	μg/m^3^	BP-3 mean 0.66 (25 %), max 3.4 BP-4 mean 1.8 (10 %), max 21.3
Jurado et al. (2014)	BPs, Beson River Delta zone	μg/m^3^	BP-1 mean 1.9 (17 %), max 19.4 BP-3 mean 0.64 (17 %), max 4.4 BP-4 mean 3.8 (25 %), max 36.6 4HB mean 0.29 (8 %), max 3.5
Surface water			
Balmer et al. (2005)	BP-3, Swiss Lakes	μg/m^3^	<2–35 Mean 16.1
Jeon et al. (2006)	BPs, rivers and lakes, South Korea	μg/m^3^	BP <25 BP-3 <25 BP-1 47 (4 %) BP-6 <25 BH <25 4HB 85 (17 %) THB <10
Pojana et al. (2007)	BP, Venice Lagoon, Italy	μg/m^3^	<2.6–136 Mean 30
Kasprzyk-Hordern et al. (2008)	BPs, river Taff, UK	μg/m^3^	BP-1 <0.3–17 BP-2 <0.5–284 BP-3 <15–44 BP-4 <3–371
Kasprzyk-Hordern et al. (2008)	BPs, river Ely, UK	μg/m^3^	BP-1 <0.3–13 BP-2 <0.5–26 BP-3 <15 BP-4 <3–323
Fent et al. 2010	BP-3, river Glatt, Swiss	μg/m^3^	56–68
Yoon et al. (2010)	BP, Han River, South Korea	μg/m^3^	<50–59 Mean 52 (33 %)
Yoon et al. (2010)	BP, effluent-dominated creeks discharging into Han River, South Korea	μg/m^3^	56–130 Mean 102
Kameda et al. (2011)	BP, rivers and lakes, Saitama Prefecture, Japan	μg/m^3^	1–68 Mean 32.2
Kameda et al. (2011)	BP-3, rivers and lakes, Saitama Prefecture, Japan	μg/m^3^	2–12 Mean 7
Rodil et al. (2012)	BP-4, rivers in Galicia, Spain	μg/m^3^	2.5–70 Mean 25
Grabicova et al. (2013)	BP-3, recreational areas (ponds, rivers) in South Bohemia, Czech Republic	μg/m^3^	12–550
Grabicova et al. (2013)	BP-4, recreational areas (ponds, rivers) in South Bohemia, Czech Republic	μg/m^3^	4.0–390
Kim and Choi (2014)	BP-3, freshwater, worldwide	μg/m^3^	<0.3–125
Food			
Balmer et al. (2005)	BP-3, fish from Swiss lakes	μg/kg f.w.	0.49–3.3 Mean 1.17
Gago-Ferrero et al. (2013)	BP-3, Guadalquivir River, Spain	μg/kg d.w.	<10–24.3 Median 20.4

#### BPs in soil and sediments

Concentrations of BP types in soils range between 0.07 and 16.55 μg/kg d.w. Sánchez-Brunete et al. (2011) investigated the concentration of different BPs in soil samples from two agricultural fields and from one industrial site in Spain. Only BP-1 was found at 5.7 μg/kg in the industrial soil and BP-6 at 0.6 μg/kg in the agricultural soil amended with sewage sludge. In Jeon et al. (2006), the parent compound BP among seven UV filters showed both high concentration (approximately 5 μg/kg) and frequency in ground soil; BP-3 was detected in 5 soil samples out of 33, at concentrations between 0.73 and 3.88 μg/kg d.w. Due to the importance of adsorption on solid matrices, more investigations would be required; for soil, only few studies are reported in the literature, so it is difficult illustrate the real presence of BPs.

Concentrations of BPs in sediments vary between 0.1 and 110 μg/kg d.w., containing BP-3 between 0.27 and 4.7 μg/kg d.w. Samples of sediments were collected from aquatic ecosystems highly urbanized and industrialized in Songhua River (China), Saginaw River and Detroit River (USA), Magdalena River (Colombia), and Biobio region (Chile) (Zhang et al. 2011; Barón et al. 2013). Nineteen sampling stations in Chile were located close to some important discharges of chemicals, food processing, and urban discharges and 13 sampling stations in Colombia included natural and urban areas of Barranquilla City that has an important industrial district and a sea-river port. Highest levels of BP-3 were detected in Colombia; the contrast with the levels found in Chile is explained with the difference in solar radiation levels. In fact, especially in Caribbean Colombian beaches, high tourism activity and use of personal care products could justify the higher BP-3 concentrations in water compared to the Chilean case (Barón et al. 2013). Kameda et al. (2011) measured BP concentration in sediments collected from rivers (polluted by industrial and domestic wastewaters) and lakes (used as the background sites) in Saitama Prefecture, Japan. Values between 2.7 and 58 μg/kg d.w. were found at the polluted sites and between 3.8 and 105 μg/kg d.w. at the background sites. Dry and wet deposition from atmosphere and the recreational activities were thought to be the source of BP-3 at the background sites. Jeon et al. (2006) measured concentrations of different UV filters in sediment samples collected from lakes and rivers in Korea affected by municipal and recreational wastewaters. The parent compound BP was the most detected and had the highest concentrations (1.52 to 9.73 μg/kg d.w.) among the investigated BPs; BP-3 was not detected in any sediment sample. Sánchez-Brunete et al. (2011) analyzed sediments collected in rivers and along the Mediterranean coast in Spain at bathing or recreational areas. BP-6 was at values between 1.2 and 6.1 μg/kg d.w. The authors that compared concentrations of sediments and surface water samples from the same location noticed that BPs in water often appeared to be below the quantification limit: most of contaminants were accumulated in sediment (Jeon et al. 2006; Kim and Choi 2014). The contamination of sediments may pose an unacceptable risk to aquatic organisms, which tend to bioaccumulate the molecules, and to humans through the ingestion of contaminated fish (Barón et al. 2013).

#### BPs in groundwater and surface water

Groundwater can be an important water supply resource; however, only few information, reported in this review, on contamination of BPs are available. Stuart et al. (2011) monitored BP concentration in groundwater in England, resulting in values between 0.03 and 2.8 mg/m^3^. Jurado et al. (2014) evaluated the presence of BPs in the Barcelona aquifers (water is used for street cleaning and to irrigate city gardens) for the first time; BP-3 was the most detected with frequency of 32 % of the samples (other compounds with frequency less than 16 %). The area under study had presented different levels in terms of concentrations; hot spot concentrations were 19.4 μg/m^3^ (BP-1), 19.2 μg/m^3^ (BP-3), and 36.6 μg/m^3^ (BP-4). Due to large amounts of WWTP effluents, Besòs River Delta was found to be the most polluted in terms of UV filter compounds. Under different redox conditions, BPs could be removed (Jurado et al. 2014); in fact, concentrations in groundwater were lower than those expected from mixing balance of the recharge sources that contributed in Barcelona aquifers.

Concentrations of BPs in surface waters are reported between 0.3 and 550 μg/m^3^. Wastewater is the most important source (Zenker et al. 2008; Fent et al. 2010; Rodil et al. 2012; Jeon et al. 2006); levels decreased with higher river water flow. Balmer et al. (2005) detected concentrations of BP-3 above the detection limit (2 μg/m^3^) in water samples from four Swiss lakes out of five (10–35 μg/m^3^); these lakes were selected because of the near municipal wastewater plants or the recreational activities. Comparable level was detected in river Glatt and lake Greifen catchment in Switzerland (Fent et al. 2010). River Glatt, near the city of Zurich, was heavily impacted by human activities and wastewater effluents of 160,000 inhabitants. The relative amount of treated wastewater in the river water was about 10–20 %. Rodil et al. (2012) measured the concentration of many emerging pollutants (including BP-3 and BP-4) in water samples collected in the metropolitan area of La Coruna (Spain). BP-4 was detected in 75 % of the surface water samples at concentrations between 2.5 and 70 μg/m^3^. BP-4 was also detected in several drinking water samples, up to a maximum level of 62 μg/m^3^. BP-3 was not found in samples of surface or drinking water. Yoon et al. (2010) investigated the occurrence of wastewater-derived contaminants including BP in water samples collected along the Han River, near the northwest Seoul metropolitan area. BP was detected in six sampling sites out of ten, at concentration between 50 and 130 μg/m^3^. Kameda et al. (2011) measured the concentrations of BP and BP-3 in water samples at polluted and background sites in the Saitama Prefecture (Japan). Values in the range 2–68 μg/m^3^ and 1–57 μg/m^3^ were found for BP and 4–12 μg/m^3^ and 2–10 μg/m^3^ for BP-3. Direct inputs (due to removal from the skin during recreational activities) may not be negligible (Kim and Choi 2014); Grabicova et al. (2013) investigated the occurrence of BPs in water samples from ponds and rivers near recreational area in the South Bohemia, comparing them with unpolluted background areas. The concentrations of both BP-3 and BP-4 in the recreational areas (12–550 and 4.0–390 μg/m^3^, respectively) were higher than those in the background areas (14–20 and 3.4–37 μg/m^3^, respectively).

#### BPs in biota and food

Few cases are available in the literature on BPs in food or aquatic biota. Balmer et al. (2005) detected BP-3 in fish from three Swiss lakes out of five; in particular, roach fish had values between 0.66 and 3.31 μg/kg f.w. Results on fish samples (common carp and Andalusian barbel) collected along the Guadalquivir River basin (Gago-Ferrero et al. 2013) confirmed the bioaccumulation of BP-3. More investigations are necessary; a limit concentration of 0.6 mg/kg in food was set for the sum of BP and 4-methylbenzophenone (IARC 2010).

## Benzotriazoles (BTs)

BTs are bicyclic heterocyclic compounds containing three nitrogen atoms and a fused benzene ring. 1H-benzotriazole (BT, C_6_H_5_N_3_, Table 1) is the reference compound of the group because it is the most used and measured in the environment compartments (DECOS 2000; Hart et al. 2004).

BTs are used as additives in fluids to inhibit corrosion of cooling systems and metal machines and in defrosting liquids (DECOS 2000); in particular, the use for defrosting purpose for aircrafts causes significant release on soils (Cancilla et al. 2003; Jia et al. 2006). Some BTs are present in pesticides and herbicides, like UV stabilizers also used in plastic devices to prevent yellowing and degradation of the products (Bhhatarai and Gramatica 2011; Wolschke et al. 2011; Zhang et al. 2011). The global production is high, about 9000 t/year in the USA (Liu et al. 2011a).

### Toxicity

Although clear antiestrogenic activity of BTs was demonstrated in vitro, no evidence of antiestrogenic activity was observed in vivo assays (Harris et al. 2007). A subchronic, 21 day reproduction toxicity tests using *Daphnia magna* resulted in a no observed effect concentration of 3 g/m^3^; based on these results, a predicted no effect concentration of 0.06 g/m^3^ was calculated (Breedveld et al. 2002). BT was classified as toxic to aquatic organisms and can cause long-term adversary effects in the aquatic environment, but it has low toxicity to humans (La Farré et al. 2008; Breedveld et al. 2002). Limited ecotoxicological data are available, mostly from acute toxicity tests on aquatic species. The EC_50_ values for fish and bacteria are 130 and 41 g/m^3^, respectively (Hem et al. 2003). Based on its molecular weight and partition coefficient, dermal absorption might be expected. Contact dermatitis was observed in metalworkers after skin exposure to BT. Based on acute toxicity data in rats (inhalation LC_50_ 2153 mg/m^3^; oral LD_50_ 500–965 mg/kg), BT should be classified as harmful for inhalation and oral exposure (DECOS 2000).

No occupational exposure limits/standards for BT have been established or recommended (DECOS 2000). USEPA (2010c) calculated a RfD of 0.03 mg/kg/day for both dermal contact and inhalation. As for carcinogenicity, based on studies in rats and mice, BT was classified as a suspected carcinogen.

### Environmental fate and transport

The discharge of treated municipal wastewater is the greatest potential source for BTs in the environment; nevertheless, overruns of wastewater sewers and atmospheric deposition can be regarded as other possible input sources (Kiss and Fries 2009).

BT has high water solubility (Table 2) and is less sorbable on organic matter than the other emerging contaminants considered in this review (Giger et al. 2006). No sorption of BTs was observed in sandy soils, while peat and compost exhibited a certain affinity with the compounds (Breedveld et al. 2002). BT has a weak hydrophobic nature (Cornell et al. 2000 in Breedveld et al. 2002) and is a weak organic acid with p*K*a of 8.6 (Andreozzi et al. 1998 in Breedveld et al. 2002).

BTs are slightly sensitive to light and photodegradable (Andreozzi et al. 1998). In Hem et al. (2003), approximately 65 % abatement of BTs was achieved at a dose of 320 mWs/cm^2^. Benitez et al. (2013) suggested a first-order kinetic for the photodegradation process in water, dependent on the pH value.

BTs are generally resistant to biodegradation; therefore, they are highly persistent in the aquatic environment (Hogenboom et al. 2009; Liu et al. 2012b). Many studies reported no evidence of microbial degradation of BT and its derivatives. In Liu et al. (2011a), BT half-lives under different redox conditions were between 114 days (aerobic conditions) and 315 days (sulfate-reducing conditions). Breedveld et al. (2002) observed no degradation during a 5 month test under anaerobic conditions, while BT removal under aerobic conditions was ascribed to evaporation due to aeration rather than biodegradation.

### Degradation byproducts

A UV degradation study of BT in aqueous solutions (Hem et al. 2003) evidenced the formation of aniline (1 % *w*/*w* of photodegradation byproducts), phenazine (10–20 % *w*/*w*), and other unknown byproducts (89–79 % *w*/*w*). The amount of byproducts increased with the UV dose. Irradiation of BT leads to two different routes of degradation: nitrogen elimination (with the production of aniline) followed by hydroxylation, and dimerization (phenazine was the main byproduct) (Benitez et al. 2013).

In Liu et al. (2012b), the degradation of four BTs in water samples was studied in aerobic and anaerobic microcosms, by using activated and digested sludge from a wastewater treatment plant. Five degradation byproducts were identified under aerobic conditions (phenol, phthalic acid, 1-methylbenzotriazole, 4-methoxy,1H-benzotriazole, and 5-methoxy,1H-benzotriazole) and four degradation byproducts (phenol, 1-methylbenzotriazole, dimethyl benzylamine, and carbazole) under anaerobic conditions.

### Environmental evidence

Table 6 summarizes the major studies on BT concentrations in different environmental matrices. WWTP effluents are important source of BTs and important direct impact is due to deicing activities. BT was detected in water and soil samples from airports, especially during the deicing season; concentrations are greatest just after the spring snowmelt (Loos et al. 2010; Breedveld et al. 2002; Heeb et al. 2012).

**Table 6:** BTs concentration in various environmental matrices

Reference	Location	Units	Value
Soils			
Breedveld et al. (2002)	Oslo Airport, Fornebu, Norway	μg/kg d.w.	100–1700 Mean 500
McNeill and Cancilla (2009)	Three USA airports	μg/kg d.w.	<3.1–4.1
Sediments			
Breedveld et al. (2002)	Oslo Airport, Fornebu, Norway	μg/kg d.w.	<100–13,000 Mean 4500
Zhang et al. (2011)	Songhua River, China	μg/kg d.w.	0.385
	Saginaw and Detroit rivers, USA	μg/kg d.w.	0.424–33.4 Mean 9.43
Groundwater			
Breedveld et al. (2002)	Oslo Airport, Fornebu, Norway	mg/m^3^	1.2–1100 Mean 371
	Oslo Airport, Gardermoen, Norway	mg/m^3^	0.11–20 Mean 4.75
Kahle et al. (2009)	Canton of Zurich, Switzerland	mg/m^3^	0.016–0.077 Mean 0.047
Loos et al. (2010)	Europe	mg/m^3^	<0.001–1.032 Mean 0.024
Liu et al. (2011b)	Next to a wastewater treatment plant, Adelaide, Australia	mg/m^3^	0.280 ± 0.018
Reh et al. 2013	Karstified aquifer, Germany	mg/m^3^	0.0049–3.2418 Median 0.0434
Surface Water			
Breedveld et al. (2002)	Oslo Airport, Fornebu, Norway	mg/m^3^	1.5–33 Mean 9.0
Weiss and Reemtsma (2005)	Lake Tegel, Berlin region, Germany	mg/m^3^	0.9
Giger et al. (2006)	Rivers in Zurich District, Switzerland	mg/m^3^	0.06–6.3 Mean 0.94
	Lake in Zurich District, Switzerland	mg/m^3^	0.02–1.2 Mean 0.55
Vousta et al. (2006)	Glatt river, Switzerland	mg/m^3^	0.636–3.69
Kahle et al. (2009)	Lakes in the Midland region, Switzerland	mg/m^3^	0.011–0.917 Mean 0.211
Kiss and Fries (2009)	Main, Hengstbach, and Hegbach rivers, Germany	mg/m^3^	0.038–1.47 Mean 0.35
Nodler et al. (2011)	Leine River, upstream of a wastewater treatment plant, Germany	mg/m^3^	0.034–0.176 Mean 0.095
	Leine River, downstream of a wastewater treatment plant, Germany	mg/m^3^	0.248–0.845 Mean 0.510
Heeb et al. (2012)	Haihe River, China	mg/m^3^	0.5–4.5 Median 1.09
Esteban et al. (2014)	Manzanares and Jarama rivers, Spain	mg/m^3^	0.097–1.184

#### BTs in soil and sediments

Few data are available on BT soil and sediment contamination; the ranges of concentration are 3.1–1700 and 0.4–13,000 μg/kg, respectively. McNeill and Cancilla (2009) investigated soils at three different US airports, but BT was detected just in one sample (4.1 μg/kg d.w.). In the old Oslo airport (Fornebu) (Breedveld et al. 2002), BT was detected in 19 out of 20 topsoil samples beside the runway, with an average concentration of 0.33 mg/kg d.w. A soil sample taken at 1.2 m depth had a concentration of 0.51 mg/kg d.w.

Breedveld et al. (2002) observed high concentrations of BT (13,000 μg/kg d.w.) in a sediment sample of a small drainage ditch draining the snow disposal site of the Fornebu Oslo airport. BT was detected at the average concentration of 420 μg/kg d.w. in three surface sediment samples and not detected (<100 μg/kg d.w.) in three other samples collected at a depth of 0.1 m in the upper organic layer of a wetland area receiving the drainage water from the snow disposal site. Regarding sediment from industrialized areas, Zhang et al. (2011) detected BT just in one sampling location (out of six) at a value of 0.39 μg/kg d.w.; on the contrary, BT was detected in all sediment samples from the Saginaw River and the Detroit River (USA) at values between 0.424 and 33.4 μg/kg d.w.

#### BTs in groundwater and surface water

BT concentrations in groundwater range between <0.001 and 1100 mg/m^3^, but the values measured by Breedveld et al. (2002) at the Oslo airports were 3 orders of magnitude higher than the maximum value measured by other authors. Excluding this paper, BT in groundwater ranges between <0.001 and 3.242 mg/m^3^. Breedveld et al. (2002) detected concentrations of BT between 1.2 and 1100 mg/m^3^ and between 0.11 and 20 mg/m^3^ in groundwater samples from the old (Fornebu) and new (Gardermoen) Oslo airports, respectively. Groundwater samples were taken from the snow disposal site, the drainage ditch, the deicing pad, and the regeneration plant of aircraft deicing/anti-icing fluids. The highest values were measured in groundwater samples from the sand and gravel deposits that were used to backfill the deicing pad during construction. The areas with the original peat and clay deposits had the lowest BT values.

Loos et al. (2010) performed a survey at European scale; BT was detected in 53 % groundwater samples collected at contaminated sites. The maximum concentration was of 1.032 mg/m^3^ and the average of 0.024 mg/m^3^. Kahle et al. (2009) detected BT in four out of six groundwater pumping stations located in a densely populated area of the Canton of Zurich (Switzerland); the detection occurred in the aquifers affected by significant water infiltration from rivers receiving considerable discharge from WWTPs. Reh et al. (2013) measured significant BT concentrations in 67 out of 163 groundwater samples collected from a karstified aquifer in Germany under an urban area of about 65 km^2^; this area was characterized by intensive industrial activities and several waste disposal sites.

In South Australia, Liu et al. (2011b) measured four BTs in groundwater collected near a WWTP, and BT was at a concentration of 0.280 ± 0.0175 mg/m^3^. The presence of the BTs in groundwater was expected due to pumping of WWTP effluents into aquifer for water reuse scheme. Often BTs were detected both in surface water and in aquifer system; in this cases, it seemed that BTs were partially eliminated through soil and subsurface passage (Kahle et al. 2009).

The concentrations of BT in surface water are reported between 0.011 and 33 mg/m^3^. Breedveld et al. (2002) measured concentrations of BT between 1.5 and 33 mg/m^3^ in samples collected in the area of the Fornebu Oslo airport. Giger et al. (2006) and Vousta et al. (2006) investigated the Glatt River and its valley, a densely populated region of 260 km^2^ with 240,000 inhabitants in the northeastern part of Switzerland. The Glatt River catchment included the northern part of the city of Zurich; ten municipal wastewater treatment plants discharged their final effluents into the river. Furthermore, the international airport of Zurich was located on the east of the Glatt River. Similar values were found in the two studies (0.06–6.3 mg/m^3^ in Giger et al. (2006) and 0.636–3.69 mg/m^3^ in Vousta et al. (2006)). The mass balance clearly indicated the input from the Zurich airport (28 % of the total BT load), where BT was used as an anticorrosive component. Giger et al. (2006) also analyzed waters from three prealpine lakes in the Canton of Zurich (Greifensee, Zurich, and Geneva). Greifensee lake is located northeast of Zurich and outflows into the Glatt River; a population of about 100,000 inhabitants live in the catchment discharging to the Greifensee. Eight municipal WWTPs discharged their treated effluents into Lake Zurich, while Lake Geneva received wastewaters from Lausanne City. BT concentrations measured in the three lakes were 0.9–1.1, 0.12–0.4, and 0.18–0.2 mg/m^3^, respectively. Kahle et al. (2009) measured BT concentration in water samples from eight lakes of the Swiss Midland region at values between 0.11 and 0.917 mg/m^3^; the BT concentrations indicate that this compound, regularly discharged to surface waters, was suitable quantitative markers of domestic wastewater in surface waters. Nodler et al. (2011) measured an increase of BT of 1 order of magnitude between water samples collected upstream (0.034–0.0176 mg/m^3^) and downstream (0.248–0.845 mg/m^3^) of the WWTP discharge in the Leine River (Germany). Kiss and Fries (2009) assessed the occurrence of BT in three German rivers (Main, Hengstbach, and Hegbach). Main and Hengstbach rivers received effluents from domestic WWTPs and were influenced by the Frankfurt International Airport; the concentrations ranged between 0.025 and 1.474 mg/m^3^. Due to the absence of wastewater effluents in the Hegbach River, a significant concentration (0.038 mg/m^3^) was observed just in one sample. In surface water samples of Spain, BT hot spots were found with a maximum concentration of 1184 μg/m^3^ in Manzanares River, 345 μg/m^3^ in Jarama River, and 1120 μg/m^3^ in Ter River (Esteban et al. 2014). Heeb et al. (2012) investigated the 175 km stretch of the Wenyu River and the North Canal from Beijing to Tianjin (China), resulting in BT concentrations between 0.5 and 4.5 mg/m^3^. Wastewaters from industrial areas and from the Beijing International Airport were the most probable sources of pollution.

## Conclusions

The CECs discussed in this review are widespread in the environment due to consistent industrial production and persistence. Table 7 summarizes the concentration orders of magnitude reported in the literature for the different environmental matrices and foodstuff; Table 8 resumes the environmental quality standards worldwide for the pollutants of concern available in the literature. By comparing the environmental quality standards in Table 8 with the ranges in Table 7, BPA levels in soils and sediments are not critical, even if the lowest value proposed for BPA in solid matrix (310 mg/kg, residential soil USA) is considered, as the maximum reported values are somewhat 3 orders of magnitude lower. However, in the future, it is possible that more stringent limits are defined. The Canadian water quality objective of 5 mg/m^3^ is quite stringent as in several situations up to an order of magnitude, and higher concentrations have been reported in the literature. For the NP, rigorous limits have been set by the European Commission. In Italy also, provisional limits 50 μg/kg for residential soil and 0.3 mg/m^3^ for groundwater exist. A more stringent water quality standard of 0.04 mg/m^3^ was proposed in Canada. As the widespread presence of NP at high levels in the various media is reported, it seems quite important to continue and intensify environmental monitoring. For BPs and BTs, the proposed standards are few; therefore, further studies are necessary for defining useful screening values to compare with. At present, the worldwide measured concentrations of BPs reported in the literature are in compliance with the values proposed for BPs by USEPA in 2007 (9.3 mg/kg for soil and 152 mg/m^3^ for groundwater). Finally, significant standards for BTs were not reported in the literature; the value of 30 mg/m^3^ proposed for surface water is about 2 orders of magnitude lower than the maximum BT concentrations. As for foodstuff and drinking water, the range of BPA concentrations in water fulfills the proposed limit for drinking water (100 mg/m^3^) (Willhite et al. 2008). The concentration of BPA in food is significant, especially in canned food, but the estimated daily intake via food consumption does not exceed the proposed RfD for oral exposure of 10 μg/kg/day (Rykowska and Wasiak 2006). In general, the concentration of NP in water does not pose risk to human health, but some values exceed the proposed limits by USEPA for acute exposure (6.6 mg/m^3^) and for chronic exposure (0.7–1.7 mg/m^3^) (USEPA 2010b). NP concentrations should be monitored in the output stream of treatment plants. High NP concentrations in food have been measured, in particular in edible fish, but in general, these values do not exceed the proposed RfD for oral exposure (100 μg/kg/day) or Danish TDI (5 μg/kg/day) (Bakke 2003). No concentration limits or guidelines have been proposed for BPs and BTs in the different environmental matrices. More investigation on the occurrence of BPs in food should be useful to exclude exceeding the proposed RfD for oral exposure (10 μg/kg/day) and the concentration limit in food (0.6 mg/kg f.w.), although the daily intake might be heavily increased by dermal sorption from cosmetics (Jeon et al. 2006; IARC 2010). Particular attention should be paid to BTs because of the high values measured in soils and sediments that might lead to exceed the proposed RfD for inhalation and dermal contact (30 μg/kg/day) (Giger et al. 2006).

**Table 7:** Orders of magnitude of concentrations reported in the literature for the investigated pollutants in the different environmental matrices and food

Pollutant	Soils and sediments (μg/kg d.w.)	Groundwater and surface water (mg/m^3^)	Foodstuff (μg/kg f.w.)
BPA	10^−1^–10^2^	10^−3^–10^2^	10^−1^–10^3^
NP	10^−2^–10^4^	10^−3^–10	10^−1^–10^3^
BPs	10^−1^–10^2^	10^−3^–10	10^−1^–10
BTs	10^−1^–10^4^	10^−3^–10^3^	–

**Table 8:** Available environmental quality standards for pollutants of concern in the investigated environmental matrices

Reference	Description	Units	Value
BPA—soils and sediments			
BCLAWS (2014)	Soil—standard, agricultural, urban park, residential soil (Canada)	mg/kg d.w.	3100
BCLAWS (2014)	Soil—standard, commercial, industrial soil (Canada)	mg/kg d.w.	31,000
USEPA (2014b)	Soil—regional screening level, residential soil (USA)	mg/kg d.w.	310
USEPA (2014b)	Soil—regional screening level, industrial soil (USA)	mg/kg d.w.	4100
BPA—waters			
CMEE (1994)	Surface water—proposed water quality objective (Canada)	mg/m^3^	5
BCLAWS (2014)	Drinking water—standard (Canada)	mg/m^3^	1800
USEPA (2014b)	Water—regional screening level, tapwater (USA)	mg/m^3^	77
NP—soils and sediments			
DanishEPA (2000)	Soil—proposed quality criteria (Denmark)	mg/kg d.w.	25
CCME (2002); Alberta (2014)	Soil—quality guidelines, residential, agricultural soil (Canada)	mg/kg d.w.	5.7
CCME (2002); Alberta (2014)	Soil—quality guidelines, commercial, industrial soil (Canada)	mg/kg d.w.	14
ISS (2011)	Soil—proposed limit for residential soil (Italy)	mg/kg d.w.	0.05
ISS (2011)	Soil—proposed limit for commercial, industrial soil (Italy)	mg/kg d.w.	12.5
CCME (2002)	Sediment—quality guidelines (Canada)	mg/kg d.w.	1.4
CIRCABC (2005)	Sediment—proposed quality standard (Europe)	mg/kg d.w.	0.18
NP—waters			
ISS (2011)	Groundwater—proposed limit (Italy)	mg/m^3^	0.3
Alberta (2014)	Groundwater—tier 1 remediation guidelines (Canada)	mg/m^3^	6.6
CMEE (1994)	Surface water—proposed water quality objective (Canada)	mg/m^3^	0.04
EC (2013)	Surface water—maximum acceptable concentration, environmental quality standard (Europe)	mg/m^3^	2
European Parliament (2013)	Surface water—maximum annual average concentration, environmental quality standard (Europe)	mg/m^3^	0.3
BPs—soils and sediments			
USEPA (2007)	Soil—remediation level Superfund Swannanoa (USA)	mg/kg d.w.	9.3
BPs—waters			
USEPA (2007)	Groundwater—remediation level Superfund Swannanoa (USA)	mg/m^3^	152
BTs—soils and sediments			
No value available in the literature			
BTs—waters			
Kase et al. (2011)	Surface water—proposed maximum acceptable concentration, environmental quality standard	mg/m^3^	120
Kase et al. (2011)	Surface water—proposed maximum annual average concentration, environmental quality standard	mg/m^3^	30

Further research should be carried out on these CECs. Treatment technologies for waters have to improve the removal efficiency of BPA and NP in order to reduce their discharge in water bodies from wastewater treatment plants. More investigation should be carried out to assess the presence and distribution of all these CECs in soils and of BPs and BTs in both surface water and groundwater. Specific studies should be carried out about possible pollution of soils due to the use of biosolids and/or treated wastewater in agriculture practices, in particular to understand the transport of CECs in the edible parts of plants and the risk for humans due to their consumption. The packaging composition for foodstuff should be reformulated to avoid contamination from BPA, NP, and BPs; moreover, the use of BPs as a flavor additive should be avoided. The knowledge of the toxicology of these compounds should be improved.
